# 25-Hydroxycholesterol modulates microglial function and exacerbates Alzheimer’s disease pathology: mechanistic insights and therapeutic potential of cholesterol esterification inhibition

**DOI:** 10.1186/s12974-025-03357-y

**Published:** 2025-02-25

**Authors:** Hayoung Choi, Haeng Jun Kim, Sang-Eun Lee, Hyun Ho Song, Jieun Kim, Jihui Han, June-Hyun Jeong, Do Yup Lee, Sunghoe Chang, Inhee Mook-Jung

**Affiliations:** 1 Department of Biochemistry and Biomedical Sciences, College of Medicine, Seoul National University, Seoul, 03080 Republic of Korea; 2 Convergence Dementia Research Center, Medical Research Center, Seoul National University, Seoul, 03080 Republic of Korea; 3 Department of Physiology and Biomedical Sciences, College of Medicine, Seoul National University, Seoul, 03080 Republic of Korea; 4 Department of Agricultural Biotechnology, Center for Food and Bioconvergence, Research Institute for Agricultural and Life Sciences, Seoul National University, Seoul, 03080 Republic of Korea

**Keywords:** Microglia, Disease-associated microglia, Aβ, Avasimibe, 25HC, Cholesterol

## Abstract

This study investigates the role of 25-hydroxycholesterol (25HC), a metabolite produced by cholesterol hydroxylase encoded by the *Ch25h* gene, in modulating microglial function and its potential implications in Alzheimer’s disease (AD) pathology. We demonstrated that 25HC impairs microglial surveillance, reduces phagocytic capacity, and increases the production of pro-inflammatory cytokines. *In vivo* two-photon microscopy revealed that 25HC administration diminishes microglial response to brain lesions, while flow cytometry confirmed reduced phagocytosis in both *in vivo* and *in vitro* models. Additionally, amyloid-beta (Aβ) was shown to upregulate *Ch25h* expression and elevate 25HC levels in microglia, exacerbating these functional impairments. Mechanistically, 25HC was found to enhance cholesterol esterification, disrupt cell membrane dynamics, and further reduce microglial mobility and phagocytosis. Treatment with Avasimibe, a cholesterol esterification inhibitor, restored membrane dynamics and microglial function, leading to attenuated AD pathology in a 5XFAD mouse model. These findings suggest that 25HC-induced changes in microglial function contribute to AD progression, and targeting cholesterol metabolism could offer therapeutic potential.

**Supplementary Information:**

The online version contains supplementary material available at 10.1186/s12974-025-03357-y.

## Introduction

Alzheimer’s disease (AD) is a progressive neurodegenerative disorder characterized by cognitive decline, memory loss, and behavioral changes. The hallmark pathological features of AD include extracellular accumulation of amyloid-beta (Aβ) plaques and intracellular neurofibrillary tangles composed of hyperphosphorylated tau protein. Despite extensive research, the precise mechanisms underlying AD pathogenesis remain incompletely understood, and effective therapeutic interventions are still lacking [1, 2]. Microglia, the resident immune cells of the central nervous system (CNS), play a crucial role in maintaining brain homeostasis by monitoring the neuronal environment, clearing debris through phagocytosis, and orchestrating inflammatory responses. In the context of AD, microglia are involved in recognizing and clearing Aβ aggregates; however, their functions become dysregulated as the disease progresses. Dysfunctional microglia exhibit impaired surveillance and phagocytic capacities, leading to inadequate clearance of Aβ and exacerbation of neuroinflammation, which collectively contribute to neuronal damage and cognitive decline [3–5]. Understanding the factors that modulate microglial function is therefore essential for elucidating AD pathology and developing targeted therapies.

Cholesterol metabolism has emerged as a significant factor influencing microglial function and AD progression. Cholesterol and its derivatives are integral components of cell membranes and play vital roles in cellular signaling, membrane fluidity, and lipid raft formation. Alterations in cholesterol homeostasis have been implicated in various neurodegenerative diseases, including AD [6–8]. Oxysterols, oxygenated derivatives of cholesterol, serve as important regulators of cholesterol metabolism and have been shown to influence immune responses and inflammation within the CNS. Among the oxysterols, 25-hydroxycholesterol (25HC) has garnered attention for its diverse biological functions. Synthesized by cholesterol 25-hydroxylase (CH25H), encoded by the *Ch25h* gene, 25HC is involved in regulating lipid metabolism, immune responses, and inflammatory processes [9, 10]. Previous studies have indicated that 25HC can modulate immune cell function by affecting cholesterol esterification and membrane dynamics, thereby influencing cell signaling and activity [11–13]. Elevated levels of 25HC have been reported in various pathological conditions, including inflammatory diseases and cancer [10, 14–16]. However, the specific role of 25HC in microglial function and its contribution to AD pathology remain poorly understood. Recent transcriptomic analyses have identified *Ch25h* as a differentially expressed gene (DEG) associated with disease-associated microglia (DAM) phenotypes in AD models [17]. This suggests a potential link between 25HC production and microglial dysfunction in the diseased brain. Furthermore, evidence indicates that Aβ may influence cholesterol metabolism pathways, including oxysterol synthesis, thereby potentially modulating microglial activity and contributing to disease progression [18, 19]. Despite these insights, the mechanistic relationship between Aβ-induced 25HC production, microglial impairment, and AD pathology has yet to be fully elucidated.

In this study, we investigated the impact of 25HC on microglial function and its subsequent effects on AD pathology. We examined how 25HC influences microglial surveillance, phagocytic capacity, and cytokine production both *in vitro *and *in vivo*. Additionally, we explored the role of Aβ in modulating *Ch25h* expression and 25HC production in microglia. We further delved into the mechanisms by which 25HC alters microglial function, focusing on its effects on cholesterol esterification and cell membrane dynamics. Lastly, we evaluated the therapeutic potential of Avasimibe, a cholesterol esterification inhibitor, in restoring microglial function and attenuating AD pathology in a 5XFAD mouse model. Our findings shed light on the critical role of 25HC in microglial dysfunction and suggest new avenues for therapeutic intervention targeting cholesterol metabolism in AD.

## Materials and methods

### Animals

The 5XFAD (Jax stock #006554, The Jackson Laboratory) littermate and transgenic(TG) were used. CX3CR1^GFP/GFP^ (Jax stock #005582, The Jackson Laboratory) was crossed with 5XFAD to obtain CX3CR1^GFP/+^_WT and CX3CR1^GFP/+^_TG mice. All animal care and experimental procedures were performed by the Institute of Laboratory Animal Resources and Control (IACUC) regulations, Seoul National University. 25HC (H1015, Sigma-Aldrich) was intraperitoneally injected at 10 mg/kg, and Avasimibe (PZ0190, Sigma-Aldrich) was orally administered at a concentration of 15 mg/kg. The amounts corresponding to the solvent in which each stock solution was dissolved (ethanol for 25HC, DMSO for Avasimibe) were administered to the control group. (Two photon imaging: CX3CR1^GFP/+^_WT *N* = 23, CX3CR1^GFP/+^_TG *N* = 12; *Ex vivo* brain sampling for bead uptake analysis and RNA extraction: 5XFAD_WT *N* = 29, 5XFAD_TG *N* = 14; Behavioral tests: Veh *N* = 23, 25HC *N* = 23, 25HC + Ava *N* = 19)

### Behavioral tests

Open field, novel objective recognition (NOR), and Y-maze tests were conducted by optimizing the experimental method of the previously established works [20, 21]. The detailed procedure information is described in the supplementary.

### Open field

Mice were freely released for 30 min in an open box (58 × 42 × 35 cm), and all behaviors were recorded with a camera. While the experiment is being conducted, the experimenter waits outside the experiment room while watching the video through the camera. Movements were subsequently analyzed with Ethovision XT 14 (Noldus IT) software.

### NOR (novel objective recognition)

On the first day, two identical objects were prepared in a box, and each mouse explored them freely for 10 min. On the second day, one old object was replaced with new type. The behavior of the mouse in the box is recorded for 5 min. The accumulated time showing exploratory behavior of directly contacting each object with the nose or feet is measured. The cognitive ability to discriminate new objects was calculated by substituting into the formula (New object exploration time - Old object exploration time) / (Total object exploration time). Mouse with a 0 total exploration time (25HC + Ava *N* = 1) was excluded from further analysis.

### Y-MAZE

A Y-shaped maze with a length of 40 cm, a height of 30 cm, and a width of 15 cm was used. The mouse was placed in the center of the Y-maze, and allowed to explore freely for 30 s, and then the direction of the arm coming and going was recorded for 7 min. The criterion for entering and exiting the arm was determined by whether the hind leg was crossed. The alternation index was calculated by dividing the number of times the direction of three consecutive entries did not overlap by the value obtained by subtracting 2 from the total number of entries. Mice with less than 15 or more than 70 total entry numbers or who jumped out of the maze during the experiment (Veh *N* = 3, 25HC *N* = 5, 25HC + Ava *N* = 3) were excluded from further analysis.

### Mouse cranial surgery and two-photon microscope imaging

Mouse cranial surgery (thinned skull) was performed by modifying a previously reported method [22–24]. Mice anesthetized through intramuscular injection (1.2 mg/kg) of a mixture of Tiletamine - Zolazepam (Virbac) and Xylazine (Bayer Korea) were fixed on a hot plate (37 °C; LCI). Cut and remove the scalp, and gently scrape the periosteum with a surgical blade and dry it. Under a dissecting microscope, carefully grind the skull using a dental microdrill and surgical blade to the bone thickness of about 20 μm (at -2.5 mm from bregma and 2.5 mm from sagittal cranial suture; somatosensory cortical area).

An LSM 7 MP (Carl Zeiss) microscope and, a Chameleon-Ultra II (Coherent) laser system, and a 20X water-immersion objective (W Plan-Apochromat 20x/1.0 DIC M27 70 mm; Car Zeiss) system were used. At 920 nm laser excitation, microglial GFP signals were collected through a 500–555 nm NDD filter. Fifty images of z-stack at 1 μm intervals were obtained at each 1 min for 60 min. The maximum power was irradiated to a 15 μm diameter ROI for local tissue damage. The responsiveness of microglia to the damaged area was scored based on previously reported methods [25]. In brief, an outer circle with a diameter of 140 μm and an inner circle with a diameter of 70 μm were drawn with the center of the damaged ROI as the center of the circle. The fluorescence intensity within each circle was measured over time. Calculate the responsive index (RI) by substituting the measured fluorescence intensity into the formula (inner circle fluorescence intensity (time) - inner circle fluorescence intensity (start) / outer circle fluorescence intensity (start)). Three-dimensional reconstruction and fluorescence intensity analysis were performed using Volocity software (Perkin-Elmer).

### Microglia isolation from adult mice

Isolation of microglia from adult mouse brains was performed as previously reported [25]. After mouse anesthesia and exsanguination, the dissected brain was placed in a DPBS solution mixed with 1.6 mg/100 ml DNase I (Sigma-Aldrich) and disrupted with a Dounce homogenizer (Wheaton Industries). After filtering the tissue homogenates in a 70 μm pore size strainer, use the Myelin and cell debris removal solution (#130-109-398, Miltenyi Biotec). Microglia were separated based on CD11b MicroBeads (Cat#130-093-634, Miltenyi Biotec, MACS, washed with cold PBS. Afterward, it was freshly incubated with a solution of beads to be used for FACS analysis or stored at -80 °C until the following process (e.g., extraction for RNA work, etc.).

## Cell culture

### Mouse primary cell culture

For the primary culture of mouse glial cells, the brains of postnatal day 1–2 outbred mice (Hsd: ICR (CD-1), KOATECH) were used. After dissecting the forebrains and peeling off the meninges on the cold HBSS (WelGENE), the brain was transferred to growth medium and repeatedly passed through the micro-glass pipette to disperse. The cell suspension is filtered through a 40 μm strainer and transferred to a T75 flask coated with Poly-D-lysine (PDL, Sigma-Aldrich). Replace the entire medium after one day and incubate it for 10–14 days. Astrocytes grow firmly attached to the bottom of the flask, and non-adherent microglia grow on it. Microglia were detached with the grown medium by tapping the flask physically. After the microglia were removed, the remaining attached astrocytes were detached with 0.25% trypsin solution and subcultured twice to use.

For the primary neuron, the brain of an embryonic day 16 outbred mouse was used. After dissecting the cerebrum and peeling off the meninges on cold HBSS, tissues are dissociated into single cells through papain (Worthington) treatment and pipetting. Neurobasal media-A (Gibco) culture medium supplemented with B27 (2%, Gibco), Sodium pyruvate (1:100 (v/v), Life technology), and Glutamax (1:100 (v/v), Gibco) was used. Half of the old culture medium was removed, and fresh media was added every 3 days. After 16 days, fully matured neurons were used for further experiments, such as drug treatment.

### Generation of iPSC-derived microglia

iPSC-derived microglia (iMG) were generated as described in the previous paper [26–28]. Briefly, iPSCs were differentiated into hematopoietic progenitor cells (HPCs) using the STEMdiff Hematopoietic kit (#05310, StemCell Technologies). On day 12, non-adherent HPCs were collected and transferred into an iMG differentiation medium for microglia differentiation. The iMG differentiation medium consisted of DMEM/F-12, HEPES, no phenol red (#11039-021, Gibco), 2% (v/v) insulin-transferrin-selenite (#41400-045, Gibco), 2% (v/v) B27 (#17504-044, Gibco), 0.5% (v/v) N2 (#17502-048, Gibco), 1% (v/v) GlutaMAX (#35050-061, Gibco), 1% (v/v) non-essential amino acids (#11140-050, Gibco), 400 µM monothioglycerol (#M1753, Sigma-Aldrich) and 5 µg/mL human insulin (#I2643-50 mg, Sigma-Aldrich,). The medium was added every other day and supplemented with 100 ng/mL IL-34 (#200 − 34, PeproTech), 50 ng/mL TGF-β1 (#100 − 21, PeproTech), and 25 ng/mL M-CSF (#300 − 25, PeproTech). On day 25 of iMG differentiation, 100 ng/mL CD200 (#BP004, Bon Opus Bio) and 100 ng/mL CX3CL1 (#300 − 31, PeproTech) were additionally added to the medium. Cells were used for the experiment after day 29 of iMG differentiation.

*In vitro* **drug treatment**.

Aβ_1-42_ (Cat#4061966, Bachem) was dissolved in HFIP at 1 mg/ml, sufficiently monomerized for 72 h, aliquoted, and lyophilized with SpeedVac (SPD2010, Thermofhisher). The aliquot was dissolved in anhydrous DMSO (Sigma-Aldrich) to 1 mM, diluted in a medium appropriate to be treated with cells. LPS (Cat#L6529, Sigma-Aldrich) stock solution was dissolved in DW at 1 mg/ml and treated at a 10 ng/ml concentration in the experiment. 25HC (H1015, Sigma-Aldrich) was used by dispensing a stock solution dissolved in DMSO at 1 mg/ml and diluting the mixture to a final 5 µg/ml concentration. Avasimibe (PZ0190, Sigma-Aldrich) was dissolved in DMSO at 500 µM as a stock solution and used at a final concentration of 2 µM. For the vehicle, the same solvent used in the stock solution was mixed with the culture medium in an amount corresponding to the drug treatment volume.

### FRAP (fluorescence recovery after photobleaching) imaging and analysis

Prepared PMG cultured on PDL-coated 18 mm cover glass and rinsed 2–3 times with Tyrode’s solution (136 mM NaCl, 2.5 mM KCl, 2 mM CaCl2, 1.3 mM MgCl2, 10 mM HEPES, 10 mM glucose, pH 7.4, ~ 290 mOsm). 1,1’-Dioctadecyl–3,3,3’,3’- Tetramethylindocarbocyanine perchlorate (DiI) fluorescence staining solution (#V22885, Invitrogen) known to be capable of staining lipids present throughout the cell membrane was mixed with Tyrode’s solution at a ratio of 1:200 (v/v), mixed by vigorous stirring, centrifuged at 13,000 rpm for 5 min, and only the supernatant was used. For live confocal imaging, a confocal fluorescence microscope (maintained at 37 °C, 5% CO2; A1Rsi s/Ti-E, Nikon; Plan Apo VC 60x Oil DIC N2 NA / 1.4; 561 nm diode laser), 4 images were acquired at 4-second intervals before photobleaching, then the ROI set for photobleaching was irradiated with maximum output laser for 10 s, and 91 images were taken for 3 min at 2 s intervals to observe recovery after photobleaching. Raw images were processed using ImageJ (NIH) software to obtain ROI fluorescence intensity over time after necessary processing, such as bleaching correction. The raw data were used as group-specific analysis data through non-linear standardization using the GraphPad Prism 8 (GraphPad Software) program. Each large *N* = 1 represents an independent culture batch, and the mean fitting values for a batch reflect quantitative values for at least 5–8 cells per group each.

### Phagocytosis assay

Fluoresbrite^®^ YG Carboxylate Microspheres (1.0 × 107; 3.00 μm; #17147-5, Polysciences), fluorescent beads, are prepared by opsonization with 1 ml of DMEM containing 50% FBS at 37 °C for 30 min while stirring at 1000 rpm. The opsonized bead mixture was diluted 1:10 in pre-warmed DMEM to finally make a 5% FBS-containing DMEM fluorescent bead solution at 1.0 × 10^6^/ml, then treated to cells for 1 h. In the case of adult mice *ex vivo* microglia, brain tissue dissected after PBS cardiac perfusion dissociated with a Dounce homogenizer (Wheaton Industries), removed debris with debris removal solution (#130-109-398, Miltenyi Biotec). After removing the debris, it was incubated in a fluorescent bead solution for 1 h. Fluorescent-tagged antibodies were incubation for 30 min. Dead cells were stained with a SYTOX™ AADvanced™ (#S10349, Invitrogen) and then analyzed by LSRFortessa X-20 (Becton Dickinson) system. Raw data were further analyzed using FlowJo (Becton Dickinson) software. Fluorescent antibodies used in FACS: Rat monoclonal anti-CD11b APC, eBioscience, 17-0112-81, (1:1000); PE/Cy7 anti-mouse CD45.2 Antibody, Biolegend, #109,829, (1:500). The antibody information is also described in the supplementary.

### Immunohistochemistry

4% PFA fixed brain slices were cryosectioned to a thickness of 30 μm in the coronal direction using a Leica CMI1850 Cryostat (Leica Biosystems, USA). The brain slices were post-fixed in 4% PFA solution, immersed in 70% formic acid-PBS solution for antigen retrieval, and then blocking and permeabilization with 10% serum of the secondary antibody donor animal and 0.3% Triton X100 solution. In between all processes, wash with PBS for 5 min each. Primary antibodies are diluted to antibody dilution solution (5% serum of a secondary antibody-donor animal to be used, 0.3% Triton X-100) and incubated with tissue section overnight at 4 °C. A secondary fluorescent antibody (Alexa FluorTM, Invitrogen; 1:500) is incubated at room temperature for 1 h. For Congo Red (Sigma-Aldrich) staining, a 1 mM aqueous solution of Congo Red was diluted 1:100 (v/v) in the secondary antibody staining step. Counterstaining was performed with DAPI (0.4 µg/ml, Sigma-Aldrich). Samples were imaged with an LSM700 (Carl Zeiss) confocal fluorescence microscope and quantified and analyzed using Image J (NIH) software. Primary antibodies used in ISH and IHC: Anti Iba1, FUJIFILM Wako Pure Chemical Corporation, 019-19741; IBA1 antibody/ Guinea pig monoclonal recombinant IgG, SYSY, #234 308; GFAP monoclonal antibody2.2B10, Invitrogen, #13–0300; Mouse monoclonal anti-β-Amyloid, biotin-labeled, clone: 4G8, BioLegend, 800,704; NeuN, Cell signaling technology, #24,307. The antibody information is also described in the supplementary.

### RNA in situ Hybridization with IHC

RNA-ISH experiments using RNAscope^®^ Multiplex Fluorescent v2 Assay combined with Immunofluorescence (Advanced Cell Diagnostics) were performed according to the methods provided by the manufacturer. Brain slices were cryosectioned to a thickness of 10 μm, placed on slides, and stored at -80 °C. The sample slide was fixed in 4% PFA, dried, dehydrated, then immersed in an antigen exposure solution, boiled at 95° C for 10 min to expose the antigen, and incubated with the primary antibody solution overnight at 4 °C. After, the primary antibody is sufficiently washed with a 0.1% Tween20 PBS (PBST) and then additionally fixed with 4% PFA. After protease treatment, the mouse *Ch25h* RNA probe was hybridized, followed by the signal amplification probe and fluorescence probe sequentially (40℃, HybEZ™ II, ACD). Finally, after blocking, fluorescently labeled secondary antibody and DAPI staining was performed. Fluorescence images were taken using LSM700 (Carl Zeiss) confocal fluorescence microscope equipment, and Image J (NIH) software was used for quantification and analysis. Primary antibodies used in ISH and IHC: Anti Iba1, FUJIFILM Wako Pure Chemical Corporation, 019-19741; GFAP monoclonal antibody2.2B10, Invitrogen, #13–0300. All antibody information is described in the supplementary table.

### Real-time PCR

Cells and tissues were lysed using RNasy Mini Kit (Qiagen), and RNA was extracted by walking on a column. The extracted RNA was synthesized into cDNA using MaximeTM RT PreMix (iNtRON Biotechnology). For the quantitative real-time reverse transcription PCR reaction, the KAPA SYBR^®^ FAST qPCR universal kit (KAPA Biosystems) was used, and the primer sequences are described in the supplementary.

### Western blotting

Dissociated cell or tissue specimen mixtures in a RIPA solution (Intronbio) were centrifuged at 13,000 rpm, 4° C, for 15 min, and only the supernatant was collected and used. The concentration of the obtained sample was measured through BCA quantification, and 10–15 µg of protein was used per well. Samples of the same amount of protein are loaded on a 4–12% Bis-Tris polyacrylamide precast gel (NuPAGE, Invitrogen) and then electrophoresis. Proteins on the gel are transferred to a PVDF membrane for subsequent antibody reaction. It is immersed in 5% skim milk solution and reacted at room temperature for 1 h to prevent non-specific binding. The primary antibodies were diluted 1: 1000 (v/v) in a solution containing the preservative sodium-azide and reacted at 4 °C for over 16 h. The membrane is then incubated with the HRP-conjugated secondary antibody for donor animals in the primary antibody at room temperature for 1 h. The HRP signal of the membrane was amplified with an ECL solution (West Save Gold, AbFrontier) and developed using an AmershamTM Imager600 (GE) instrument. The obtained band images were analyzed and quantified with MultiGauge software (FujiFilm). Primary antibodies used in WB: Anti Iba1, FUJIFILM Wako Pure Chemical Corporation, 019-19741; CH25H Polyclonal Antibody, Invitrogen, PA5-72349; Mouse monoclonal anti-β-actin, Sigma-Aldrich, Ab1978; Goat polyclonal anti-mouse Il-1β, R&D systems, AF-401-NA; Human/Mouse TNF-α antibody, R&D systems, AF-410-NA; Anti-PSD95 antibody, Abcam, ab18258; Anti-Synaptophysin antibody, Millipore Sigma,, MAB368; Anti-TMEM119 antibody [28 − 3], Abcam, ab209064; Monoclonal Anti-β-Tubulin I + II antibody produced in mouse, Sigma-Aldrich, T8535. Information on all antibodies used is provided in the Supplementary Materials.

### Aβ ELISA

Brain tissue stored at -80 °C after rapid cooling with LN_2_ immediately after extraction was homogenized with a PBS in a volume four times its weight. The homogenized sample was mixed with RIPA (Intronbio) solution 1:2 (v/v) and centrifuged at 13,000 rpm at 4 °C for 5 min. All procedure was performed on ice to minimize heat-induced protein damage. The concentration of the obtained brain tissue disruption solution is measured and adjusted to 1 µg/µl by adjusting the RIPA disruption solution and then ultracentrifuge at 100,000 g at 4 °C for 1 h. The supernatant obtained at this time is RIPA-soluble fraction. The remaining pellet is disrupted in 400 µl of 70% formic acid aqueous solution and ultracentrifuge at 100,000 g at 4 °C for 1 h. The formic acid soluble supernatant obtained at the end of the second ultracentrifugation is a RIPA insoluble protein fraction, which was neutralized by mixing with 1 M, pH 11 Tris buffer at a ratio of 1:20 (v/v) and used in subsequent experiments. ELISA assay was performed using Abeta(1–42) ELISA kit (#27711, IBL) according to the test method provided by the manufacturer.

## GC-MS analysis

### Metabolites extraction from primary microglia

Microglia pellets were thawed at 4 °C on ice and mixed with 1400 µL of extraction solvent consisting of methanol, isopropanol, and water in a ratio of 3:3:2 (v/v/v). The samples were homogenized using a Mixer Mill MM400 (Retsch GmbH & Co., Germany) with steel beads for 90 s and sonicated for 15 min. After centrifugation at 13,200 rpm for 5 min at 4 °C, 1300µL of each supernatant was transferred into a new 1.5-mL tube. The aliquots were concentrated to dryness in a speed vacuum concentrator (SCANVAC, Korea) and stored at -80 °C until derivatization.

### Gas chromatography time-of-flight mass spectrometric analysis

The dried extracts were subjected to derivatization using 5µL of 40 mg/mL methoxyamine hydrochloride in pyridine and incubated for methoxyamination at 200 rpm and 30 °C for 90 min. For retention time index, a mixture of fatty acid methyl esters [FAMEs] was added (2 µL), and 45 µL of N-Methyl-N-trimethylsilyltrifluoroacetamide (MSTFA + 1% TMCS; procured from Thermo) was combined for trimethylsilylation at 200 rpm and 37 °C for 1 h. The FAME mixture contained C8, C9, C10, C12, C14, C16, C18, C20, C22, C24, C26, C28, and C30.

The GC-MS analysis was performed by an Agilent 7890B gas chromatograph system (Agilent Technologies) with an RTX-5Sil MS column (Restek). The derivatives (0.5 µL) were injected in splitless mode, and the entire system was controlled using ChromaTOF software 4.50 version (LECO). Mass spectra were acquired at an acquisition rate of 20 spectra/s in the range of 85–500 m/z using a Leco Pegasus HT time-of-flight mass spectrometer. The mass-spectrometry analysis was performed in a randomized order for all samples. The raw data were aligned using MS-DIAL for further processing.

### Cellular cholesterol/cholesteryl ester assay

Using a chloroform-free extraction kit (#K216, BioVision), intracellular lipids were extracted by the instructions provided by the manufacturer. Cholesterol and cholesteryl-ester (CE) were measured using a kit (Ab65359, Abcam) based on a cholesterol-specific probe’s color development and luminescence reaction. The dried lipid extracts were dissolved in the reaction buffer provided. Load the standards and samples into a 96-well microplate and add the provided reaction mixture to measure free cholesterol and total cholesterol. After reacting for 60 min in a dark place at 37° C, an OD 570 nm measurement value is obtained using a microplate reader (Tecan). The concentration of the sample was calculated with a standard curve using the standards’ concentration and color development.

### Quantification and statistical analysis

GraphPad Prism 8 (GraphPad Software) was used for data analysis. A significant difference between two groups was calculated using a *t*-test, and a significant difference between three or more groups was calculated using a one-way ANOVA test. The microglial RI data taken with a two-photon microscope imaging was compared by slope comparison analysis calculating a linear regression equation for the corresponding raw intensity value. In the case of FRAP analysis results, average regression curve data for each group was obtained by non-linear fitting for raw intensity data. *N* of *in vitro* experiments, except FRAP experiments, means the number of experiments performed individually. For the FRAP experiment, the average of at least five or more images performed on the same day per group was *N* = 1. N in *ex vivo* and *in vivo* experiments represent the number of animals used.

### Data Availability

The data that support the findings of this study are available from the corresponding author, upon reasonable request.

## Results

### Aβ upregulates Ch25h expression and 25HC production in microglia

**Fig. 1:**
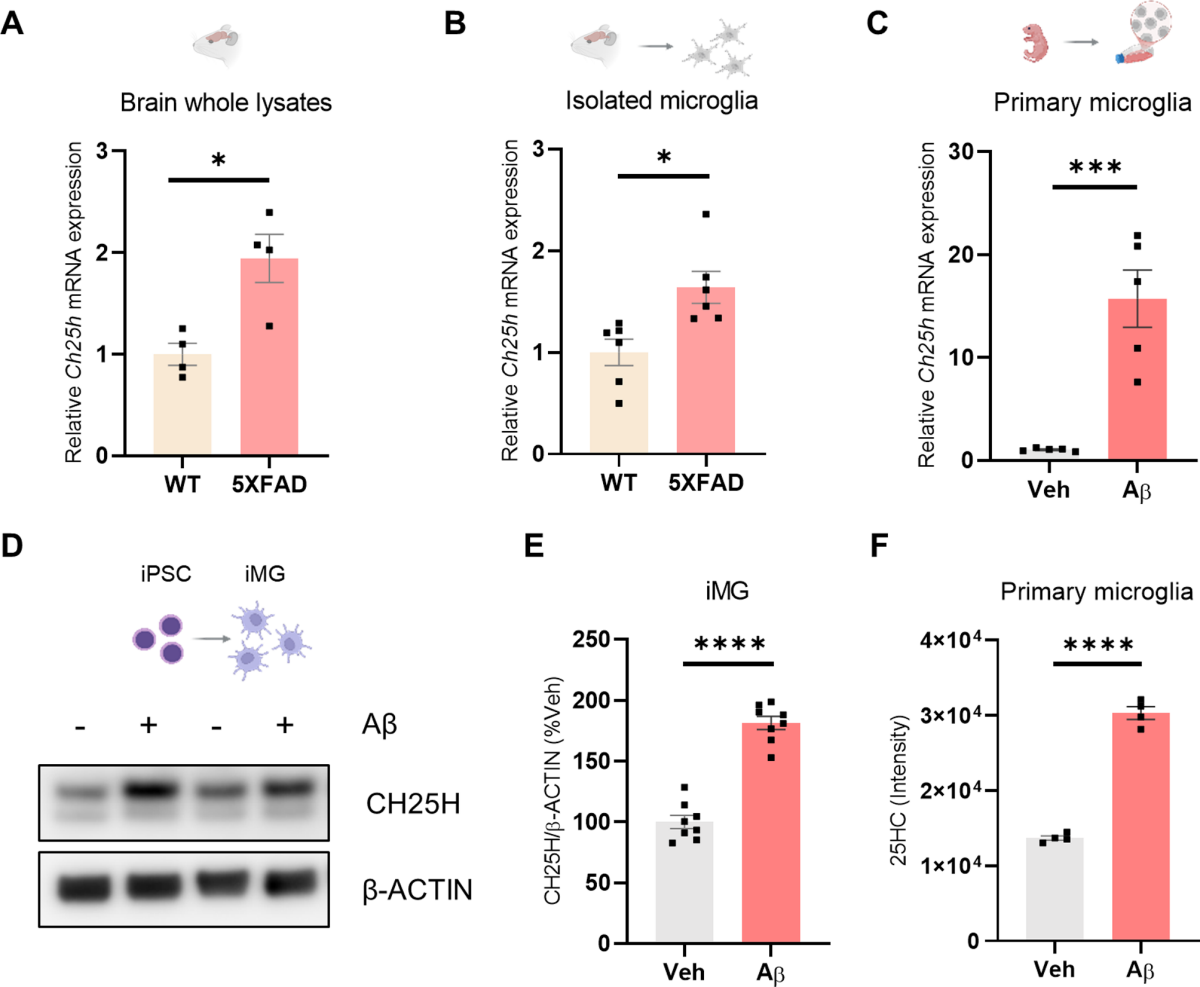
Aβ upregulates *Ch25h* expression and 25HC production in microglia. (**A**) *Ch25h* mRNA expression levels in whole brain lysates by RT-PCR (5XFAD_WT and 5XFAD_TG, 3-month-old; N=4). (**B**) *Ch25h* mRNA expression levels *ex vivo* isolated adult microglia (5XFAD_WT and 5XFAD_TG, 7-month-old; N=6). (**C**) *Ch25h* mRNA expression level after treating PMG with vehicle or Aβ (4 μM) for 24 hours (N=5). (**D, E**) The CH25H protein expression level was measured by WB after treatment of iMG with vehicle or Aβ (2 μM) for 24 hours (N=6)

Among the DEGs reported as DAM characteristics, we focused on *Ch25h*, the gene encoding a type of cholesterol hydroxylase that appears to be closely related to the immune response of microglia and changes in lipid metabolism [9, 10, 29]. First, by comparing the mRNA levels of *Ch25h* in brain tissues of the 5XFAD model, it was found that *Ch25h* expression was significantly increased in the TG group compared to WT in both whole brain lysates and isolated microglia (Fig. 1A, B). When Aβ was directly treated with primary microglia (PMG), *Ch25h* mRNA expression was significantly increased compared to the control group (Fig. 1C). An *in vitro* model of human iPSC-derived microglia (iMG) was used to confirm the change in CH25H protein levels in microglia upon Aβ stimulation, as no antibody targeting mouse CH25H is available. Aβ treatment significantly increased CH25H protein expression in iMG cells (Fig. 1D, E).

Given that *Ch25h* is not a gene that is specifically expressed in microglia, we attempted to visualize the quantity and spatial distribution of *Ch25h* expression in the brain tissue of 5XFAD mice using RNA-in situ hybridization. Most of the *Ch25h* fluorescence was observed to co-localize with IBA1, a microglia-specific protein, but there was some minor overlap with GFAP, an astrocyte-specific protein (Extended Data Fig. 1A, B). In addition, to determine whether the changes in *Ch25h* expression induced by Aβ treatment were also observed in other brain cells beyond microglia, an *in vitro* primary mouse cell culture model was used. The results showed no difference in *Ch25h* mRNA expression in primary mouse neurons in response to LPS and Aβ treatment. Similarly, Aβ treatment did not significantly affect mRNA levels in primary mouse astrocytes. Instead, only stimulation with LPS, a stronger immunogen, resulted in an approximately threefold increase in expression (Extended Data Fig. 1C-E). Taken together, it appears *Ch25h* induction by Aβ stimulation occurs in a highly microglia-specific manner. Furthermore, we tested whether the amount of 25HC, a metabolite produced by the CH25H enzyme, was altered by Aβ treatment. As a result, the GC-MS analysis of 25HC extracted from PMG lipids revealed a significant increase in 25HC levels in the Aβ-treated group compared to the control group (Fig. 1F). These findings indicate that Aβ specifically enhances *Ch25h* expression and 25HC production in microglia.

### 25HC exerts effects on microglial surveillance, phagocytosis and cytokine production

**Fig. 2:**
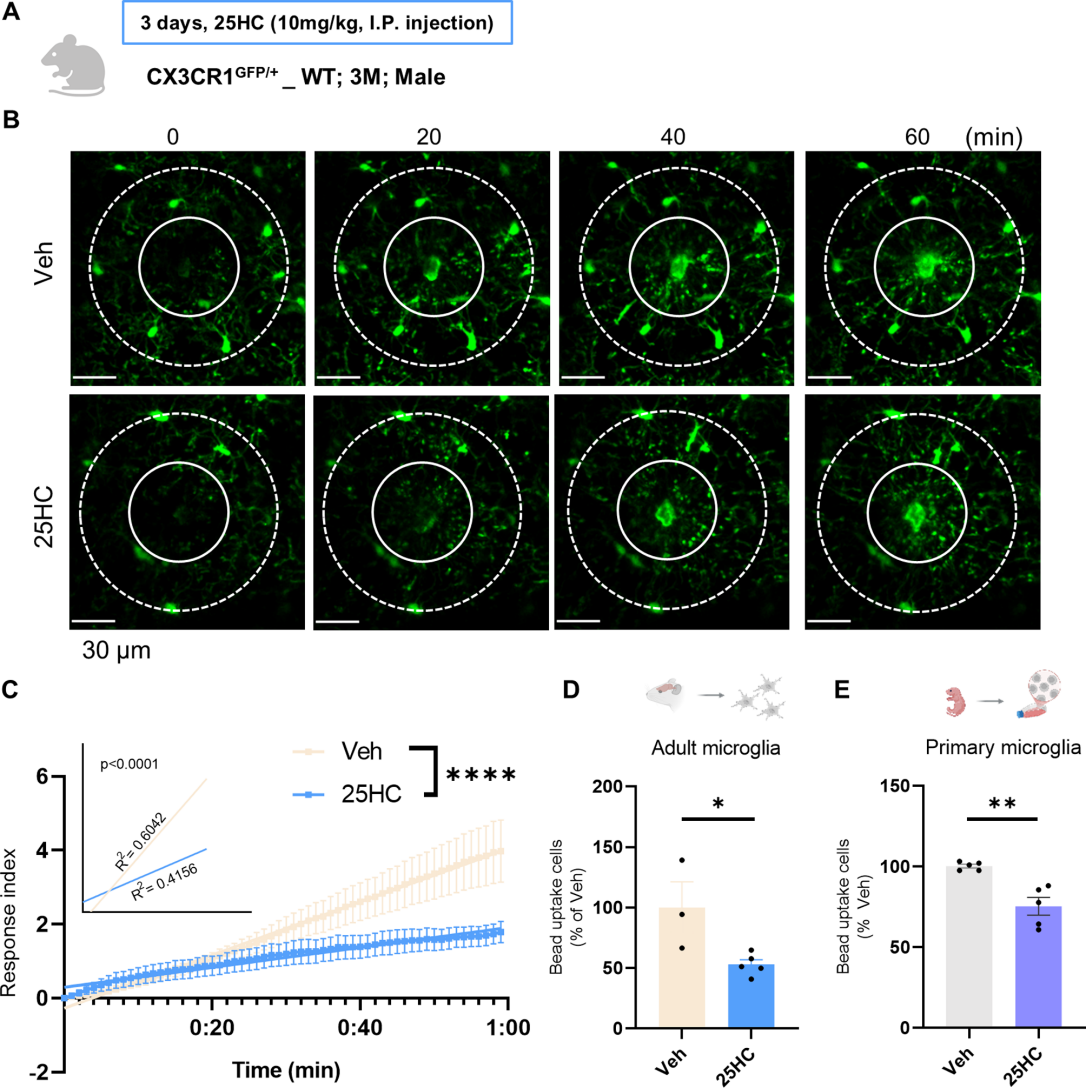
25HC reduces microglial movement and phagocytic capacity. (**A, B**) Real-time intravital imaging of two-photon microscope of CX3CR1^GFP/+^_WT mice microglia movement in response to tissue damage after intraperitoneal injection of vehicle or 25HC (10 mg/kg) and RI (3-month-old; N=5; Scale bar = 30 μm; Outer circle diameter 140 μm, inner circle diameter 70 μm). (**C**) *Ex vivo* isolated microglia bead uptake FACS analysis after intraperitoneal injection of vehicle or 25HC (10 mg/kg) to 5XFAD_WT (3-month-old; N=3). (**D**) *In vitro* PMG bead uptake FACS analysis after vehicle or 25HC (5 μg/ml) treatment for 24 hours (N=5). (Mean ± SEM; Slope comparison analysis; ∗∗∗∗p < 0.001; unpaired t-test; *p<0.05, ***p<0.005)

Next, to determine the effect of increased *Ch25h* and 25HC on microglial function, we treated 25HC directly and examined whether any changes occured in microglial surveillance function, phagocytic capacity, and cytokine secretion. The first step involved monitoring live microglia *in vivo* using a two-photon microscope. Since most oxysterols, including 25HC, are known to to have a high permeability across the blood-brain barrier (BBB), 25HC was administered via intraperitoneal (IP) injection to avoid brain damage and microglial activation that could result from direct cranial injection [30–32]. Following the administration of 25HC to CX3CR1^GFP/+^_WT mice, a model in which CX3CR1-positive microglia are labeled with GFP, focal brain irradiation was induced, and the resulting microglial movement response to the lesions was observed (Fig. 2A, Supplementary Movie 1). The microglia in the 25HC-administered group exhibited a significantly lower response index (RI) compared to the vehicle-administered group (Fig. 2B, C). The phagocytic capacity of microglia was evaluated by FACS analysis of freshly isolated microglia from drug-treated mouse brains, followed by treatment with opsonized fluorescent microbeads (Fig. 2D, Extended Data Fig. 2B). The proportion of cells that internalized fluorescent beads was significantly lower in the 25HC-treated group compared to the control group (Fig. 2D). This reduction in phagocytic activity was also observed *in vitro* using PMG (Fig. 2E, Extended Data Fig. 2C). The decline in microglial phagocytic capacity was shown to worsen as AD pathology progressed with age (Extended Data Fig. 2A). These findings suggest that 25HC may be a key factor directly contributing to the reduced phagocytic capacity of microglia in the AD brain. Additionally, it was demonstrated that 25HC treatment significantly increased the synthesis and secretion of inflammatory cytokines, including TNF-α in the cell culture medium and IL-1β within cells, both of which are associated with neurotoxicity (Extended Data Fig. 3).

Taken together, the increased *Ch25h* expression and 25HC production in microglia in response to Aβ slowed their clearance capacity and accelerated the inflammatory response, which could be detrimental to the AD brain. Considering that it was previously reported that the responses of microglia recruited to the lesions were reduced in CX3CR1^GFP/+^_5XFAD [25], it seems that the increase in microglial *Ch25h* and 25HC production by Aβ is directly related to the mechanism of Aβ-induced decrease in microglial surveillance.

### 25HC stimulates cholesterol esterification and disrupts membrane dynamics

We aimed to understand how 25HC inhibits microglial movement and phagocytic ability. As 25HC is a trace metabolite in the brain among various oxysterols [33], we focused on identifying factors that could act locally and directly on microglia. In peripheral immune system macrophages, 25HC has been reported to increase upon exposure to various infectious agents, inducing qualitative changes in cholesterol within cell membranes. These changes can suppress further entry and spread of infectious agents. Notably, previous studies have shown that 25HC enhances the production of cholesterol esters (CE) by upregulating cholesterol esterase [11]. CE produced in this manner reduces the amount of free, accessible cholesterol in the cell membrane [12, 13].

**Fig. 3:**
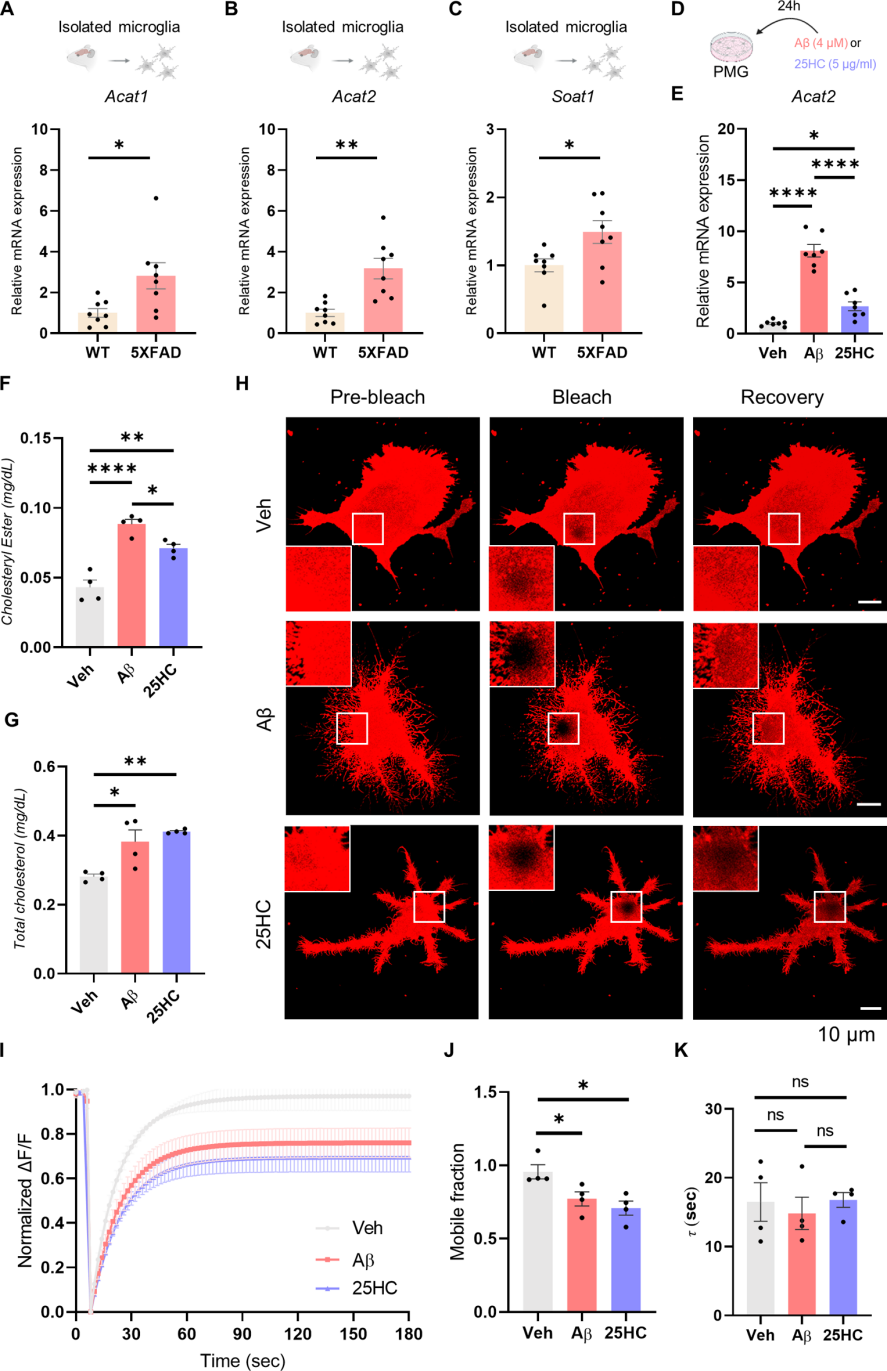
25HC stimulates cholesterol esterification and disrupts membrane dynamics. (**A-C**) *Ex vivo* isolated microglial *Acat1*, *Acat2*, and *Soat1* mRNA level measured by RT-PCR (5XFAD_WT and 5XFAD_TG, N=8; 7-month-old). (**D**) Scheme of drug administration. (**E**) *Acat2* mRNA expression level after treating PMG with vehicle or Aβ (4 μM) or 25HC (5 μg/ ml) for 24 hours (N=6-7). (**F, G**) Cholesterol and cholesteryl ester quantification from PMG lysates after treating PMG with vehicle or Aβ (4 03BCM) or 25HC (5 μg/ml) for 24 hours (N=4). (**H**) Representative images of PMG FRAP analysis after treating PMG with vehicle or Aβ (4 μM) or 25HC (5 μg/ml) for 24 hours (Scale bar = 10 μm). (**I**) Normalized graph of fluorescence intensity change of ROI from FRAP analysis (N=4). (**J, K**) Quantification of mobile fraction and τ of FRAP analysis (N=4, One-way ANOVA, Holm-Sidak multiple comparison tests; *p<0.05, ns, non-significant). (Mean ± SEM; unpaired t-test; *p<0.05, **p<0.01; One-way ANOVA, Tukey’s multiple comparison tests; *p<0.05, **p<0.01, ***p<0.005, ****p<0.001; n.s., non-significant)

We investigated whether 25HC-induced changes in cell membrane composition affect microglial movement, responsiveness to Aβ, and phagocytic capacity. First, we explored how Aβ and 25HC influence the expression of cholesterol esterase in microglia. *Ex vivo* microglia isolated from WT and TG mice of the 5XFAD model, the mRNA levels of cholesterol esterases *Acat1*,* Acat2*, and *Soat1* were significantly elevated in TG microglia (Fig. 3A-C). *In vitro*, treatment of PMG with Aβ and 25HC significantly increased *Acat2* expression (Fig. 3D, E). Similarly, *Soat1* expression was significantly elevated following direct treatment with 25HC, although no significant changes were observed for *Acat1* (Extended Data Fig. 4A, B). Considering that the products can change the level of expression of the mRNA of the enzymes, we tried to verify the actual change in the CE. A cellular CE assay revealed that CE levels in PMG treated with Aβ and 25HC were significantly higher than those in the control group (Fig. 3F, Extended Data Fig. 4C). This increase in CE was accompanied by a parallel rise in total cholesterol levels (Fig. 3F, G, Extended Data Fig. 4D, E). To determine whether increased CE impacts cell membrane dynamics, we measured membrane fluidity using FRAP experiments. Cell membranes of PMG were fluorescently labeled with DiI dye, which stains lipids. The results showed that the Aβ and 25HC-treated groups exhibited a significantly reduced mobile fraction, with weaker fluorescence recovery compared to the vehicle-treated group (Fig. 3H-J, Extended Data Fig. 4F, G). However, there were no significant differences in the recovery time constant (τ), which reflects the time required to reach saturation (Fig. 3K, Extended Data Fig. 4H). These findings suggest that Aβ and 25HC promote increased CE production in microglia. This alteration in cellular lipid composition leads to impaired cell membrane dynamics, potentially contributing to the reduced mobility and functionality of microglia.

**Fig. 4:**
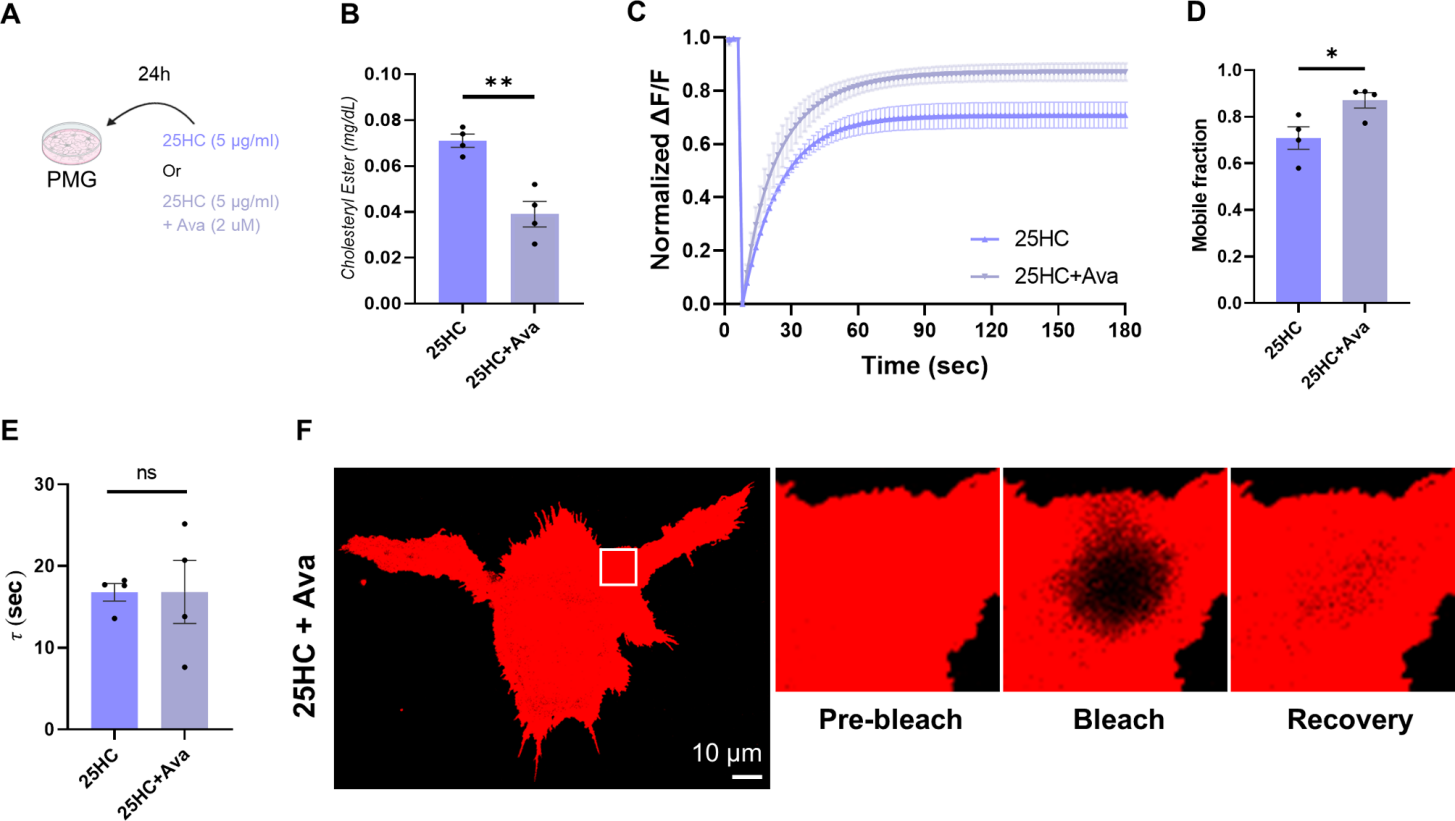
Cholesterol esterification inhibitors reduce the CE level and restore the microglial membrane dynamics. (**A**) Scheme of drug administration. (**B**) Cholesterol and cholesteryl ester quantification from PMG lysates after treating PMG with only 25HC (5 μg/ml) or with Avasimibe (2 μM) for 24 hours (N=4). (**C**) Normalized graph of fluorescence intensity change of ROI from FRAP analysis (N=4). (**D-E**) Cholesterol and cholesteryl ester quantification from PMG lysates after treating PMG with only 25HC (5 μg/ml) or with Avasimibe (2 μM) for 24 hours (N=4). (**F**) Representative images of PMG FRAP analysis (Scale bar = 10 μm). (Mean ± SEM; N=4; unpaired-t test; *p<0.05, n.s., non-significant)

### Cholesterol esterification inhibitors reduce the CE levels and restore the microglial function disrupted by 25HC

A strategy to block CE production was tested to determine if there was a link between the observed decrease in responsiveness and phagocytic capacity and changes in cell membrane dynamics caused by increased CE production. Avasimibe, a cholesterol esterification inhibitor, was used to reduce CE production. First, the cholesterol quantification experiment confirmed that treatment with Avasimibe reduced CE, which was increased by 25HC (Fig. 4A, B). Further FRAP analysis shows that when Avasimibe reduced CE, the actual inhibition of cell membrane dynamics was also restored (Fig. 4C–F, Extended Data Fig. 4F–H).

**Fig. 5:**
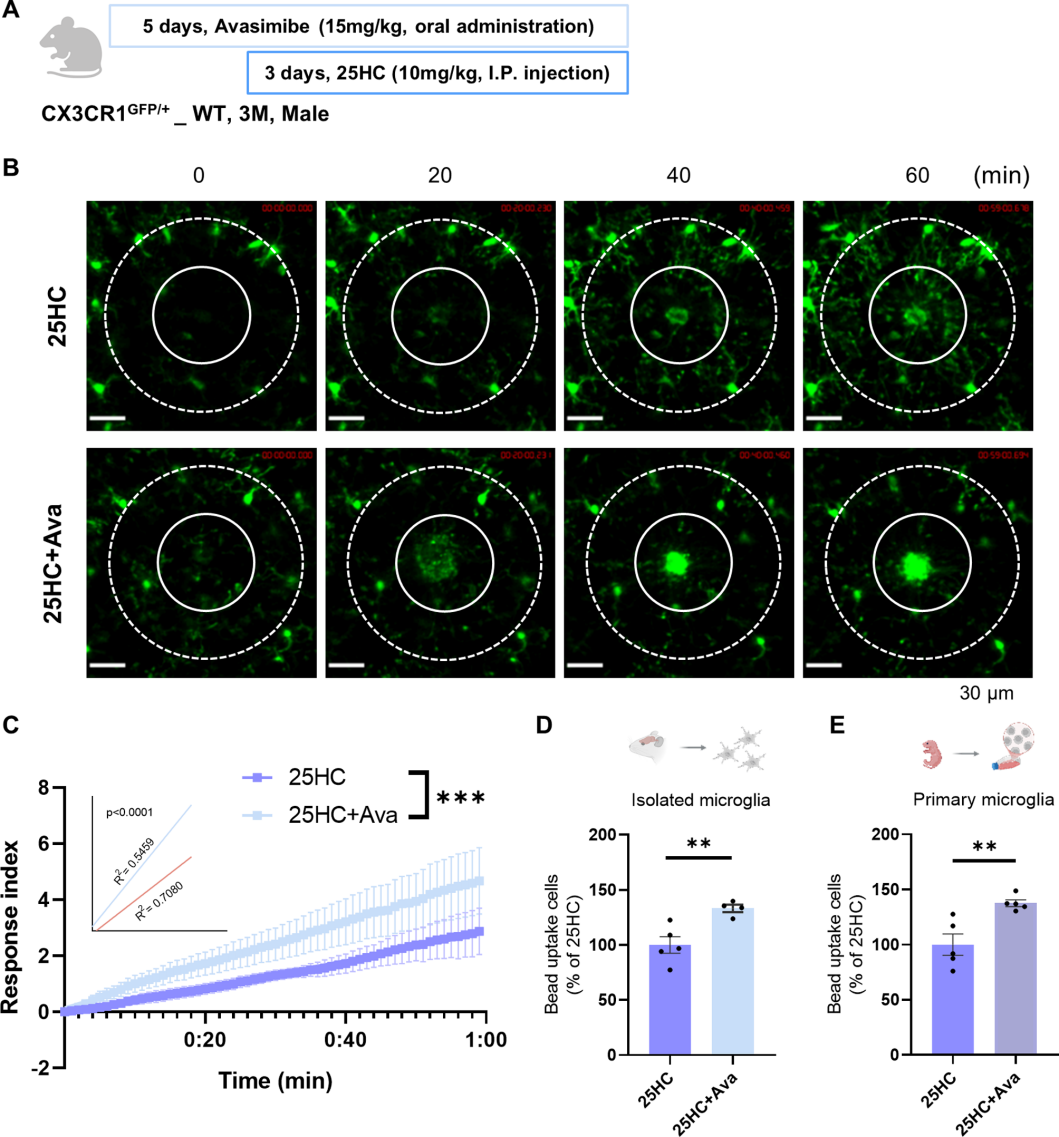
Cholesterol esterification inhibitor restores the microglial movement and phagocytic capacity. (**A**) Scheme of drug administration. (**B-C**) Real-time intravital imaging of two-photon microscope of CX3CR1^GFP/+^_WT mice microglia movement in response to tissue damage after drug treatment. Representative images by time (**B**) and quantitative graph of response index (**C**) (3-month-old; 25HC N=3, 25HC+Ava N=4; Scale bar = 30 μm; Outer circle diameter 140 μm, inner circle diameter 70 μm). (**D**) *Ex vivo* isolated microglia bead uptake FACS analysis after drug treatment (3-month-old; 25HC N=5, 25HC+Ava N=4). (**E**) *In vitro* PMG bead uptake FACS analysis after treating only 25HC (5 μg/ml) with Avasimibe (2 μM) for 24 hours (N=5). (Mean ± SEM; Slope comparison analysis; ∗∗∗p < 0.001; unpaired-t test **p<0.01)

Since Avasimibe is known to have high BBB permeability [34], it was administered to the mice by oral gavage using the previously proposed effective dosing method (Fig. 5A) [35]. The movement of microglia *in vivo*, monitored using two-photon microscopy, was compared between groups treated with 25HC alone and those treated with a combination of 25HC and Avasimibe. The results showed that co-administration of Avasimibe with 25HC restored microglial movement (Fig. 5B–C, Supplementary Movie 2). Similarly, *ex vivo* analysis revealed that the phagocytic capacity of microglia was restored when Avasimibe was administered alongside 25HC (Fig. 5D).* In vitro* experiments with PMG also demonstrated alterations in phagocytic capacity. Treatment with Avasimibe significantly increased the phagocytic ability that had been reduced by 25HC (Fig. 5E). These findings suggest that changes in cell membrane quality caused by CE are closely linked to microglial movement and phagocytosis. Furthermore, the functional impairments in microglia may result from Aβ-induced overproduction of 25HC and CE.

### 25HC accelerates AD pathology in 5XFAD and CE inhibitor attenuate them

**Fig. 6:**
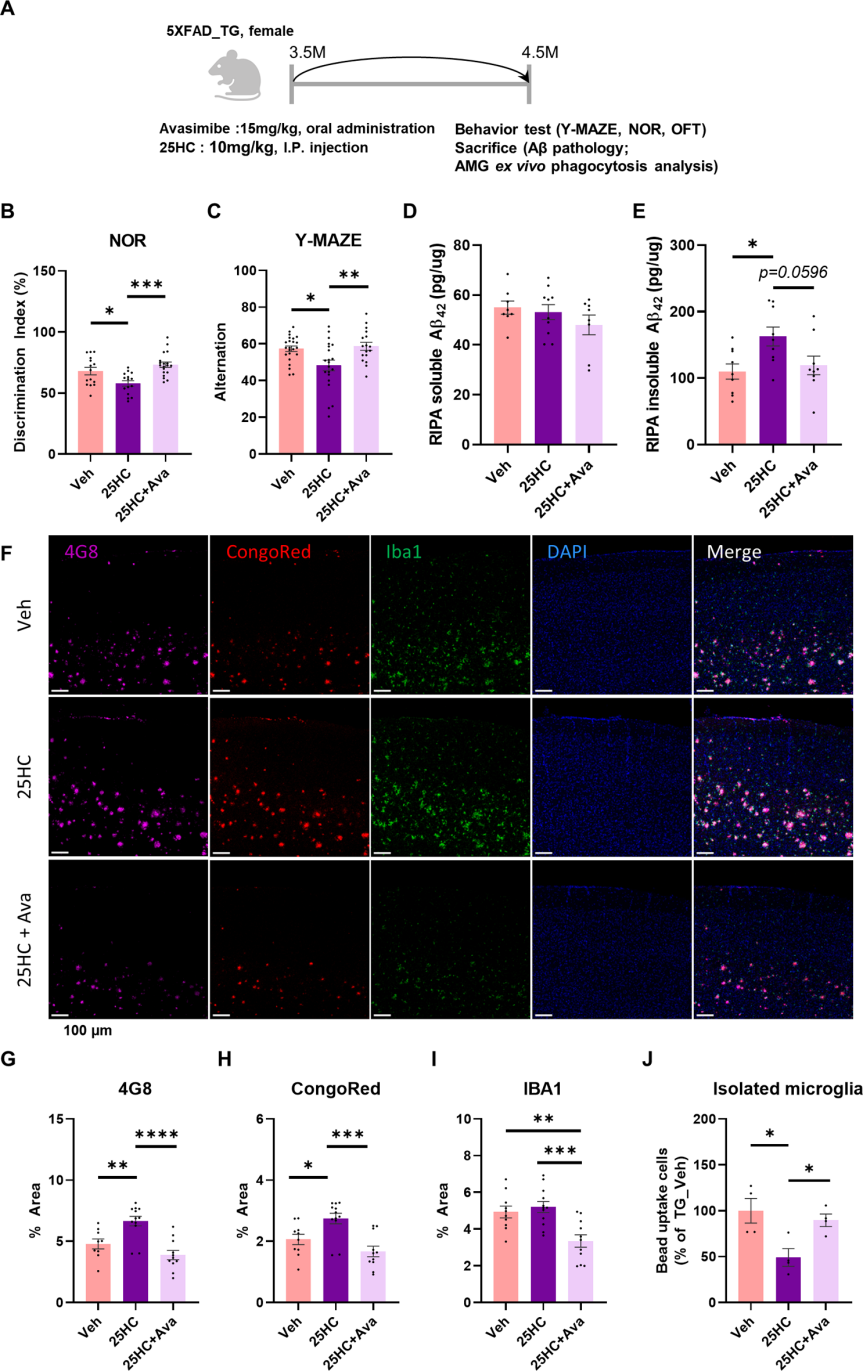
25HC accelerates AD pathology in 5XFAD and CE inhibitor attenuate them. (**A**) Scheme of 5XFAD_TG drug administration and analysis. TG: 5xFAD transgenic mouse. (**B**) Quantitative graph of discrimination index after NOR behavioral test (Veh N=15, 25HC N=15, 25HC+Ava N=19; Tukey’s multiple comparison tests). (**C**) Y-MAZE alteration rate (Veh N=23, 25HC N=19, 25HC+Ava N=17; Tukey’s multiple comparison test). (**D, E**) Quantitative results of RIPA soluble and insoluble Aβ_1-42_ present in mouse prefrontal cortex by ELISA (Veh N=8, 25HC N=10, 25HC+Ava N=8; Holm-Sidak’s multiple comparison tests). (**F-I**) Representative images and quantitative results of 5XFAD mouse cerebral cortex observed with 4G8, CongoRed, and IBA1 (Veh N=9, 25HC N=12, 25HC+Ava N=11; Tukey’s multiple comparison tests). (**J**) *Ex vivo* isolated microglia bead uptake FACS analysis after drug treatment (N=4; Holm-Sidak multiple comparison test). (Mean ± SEM; One-way ANOVA, Tukey’s multiple comparison tests; *p<0.05, **p<0.001, ***p<0.005, ****p<0.001)

**Fig. 7:**
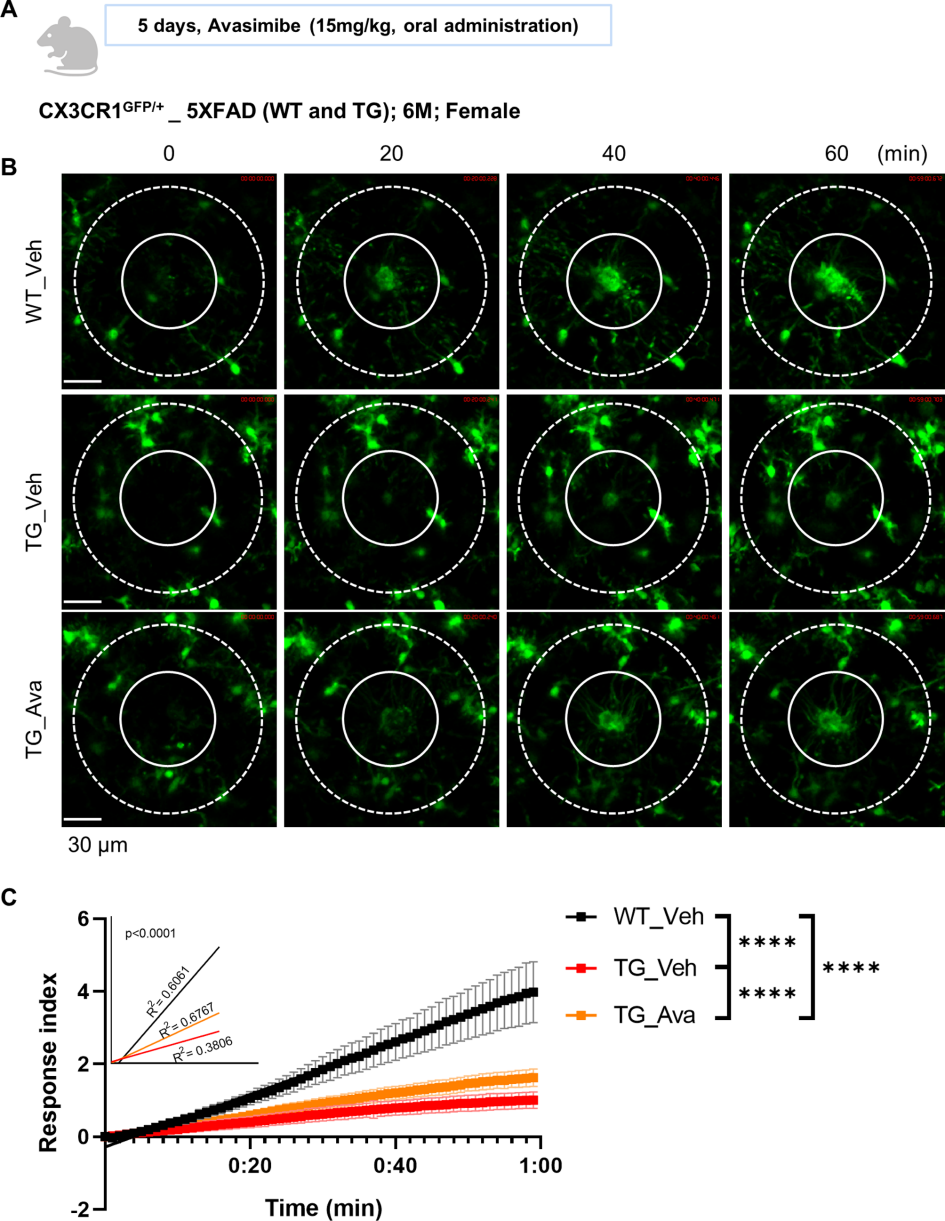
Cholesterol esterification inhibitor restores the *in vivo* microglial movement of 5XFAD_TG. (**A**) Scheme of CX3CR1^GFP/+^_5XFAD drug administration. WT: Wild type mouse; TG: 5xFAD transgenic mouse. (**B-C**) Real-time intravital imaging of two-photon microscope of CX3CR1^GFP/+^_5XFAD mice microglia movement in response to tissue damage after drug treatment. Representative images by time (**B**) and quantitative graph of response index (**C**) (N=6; Slope comparison analysis; ∗∗∗p < 0.001; Scale bar = 30 μm; Outer circle diameter 140 μm, inner circle diameter 70 μm)

We sought to determine whether microglial dysfunction induced by 25HC ultimately accelerates the progression of AD. To test this, 3.5-month-old 5XFAD TG mice, representing an early stage of pathology, were treated with Veh, 25HC, or 25HC combined with Avasimibe every other day for four weeks. Microglial function and pathological changes were then assessed (Fig. 6A). Cognitive and memory functions were evaluated using the novel object recognition (NOR) discrimination index and the Y-maze test. The 25HC-treated group performed significantly worse than the control group in both tests. However, mice treated with 25HC and Avasimibe exhibited improved cognitive function compared to the 25HC-only group (Fig. 6B–C, Extended Data Fig. 5). Long-term drug administration did not impair basic locomotor activity, as indicated by the total number of Y-maze entries and total distance traveled in the open field test. Instead, abnormal movements were observed in the 25HC-treated group, suggesting that 25HC may increase anxiety and induce abnormal behavior in mice (Extended Data Fig. 5A–C, E). Importantly, there were no significant differences in weight changes or metabolic abnormalities among the groups after four weeks of treatment, indicating no adverse side effects from long-term administration (Extended Data Fig. 5D). Microglial phagocytic capacity, assessed *ex vivo*, was significantly reduced in the 25HC-treated group but was restored with Avasimibe co-treatment (Fig. 6J). To evaluate changes in amyloid pathology, RIPA-soluble and insoluble Aβ_1−42_ levels in the prefrontal cortex and hippocampus were quantified by ELISA. The 25HC-treated group showed significantly elevated levels of RIPA-insoluble Aβ_1−42_ compared to controls, while co-treatment with Avasimibe reduced these levels (Fig. 6D, E, Extended Data Fig. 6E, F). Immunohistochemistry further revealed that the density of Aβ plaques, stained with the 4G8 antibody and CongoRed, was significantly higher in the 25HC-treated group but reduced with Avasimibe treatment, consistent with ELISA results (Fig. 6F–I, Extended Data Fig. 6A–D, G–I). IBA1 staining showed reduced microglial density in the Avasimibe-treated group, likely reflecting either reduced microgliosis or a decline in microglial numbers due to decreased amyloid pathology (Fig. 6I, Extended Data Fig. 6A, D). Similarly, IBA1 protein levels in brain lysates showed a downward trend in the Avasimibe-treated group, aligning with imaging results, though the changes were not statistically significant (Extended Data Fig. 6A, B). The amount of TMEM119 protein, a marker for homeostatic microglia, was assessed in prefrontal cortex lysates. While there was no significant difference among groups, the 25HC-treated group exhibited the greatest reduction, suggesting fewer functional microglia in brains with accelerated plaque pathology (Extended Data Fig. 7A, C). Consistent with this, astrogliosis was reduced in the Avasimibe-treated group. However, contrary to expectations based on the dramatic behavioral differences, NeuN staining and the expression of synaptic markers such as PSD95 and synaptophysin in prefrontal lysates showed no statistical differences between treatment groups (Extended Data Fig. 7A, D–K). *In vivo* two-photon imaging was used to assess whether Avasimibe directly improved microglial function in aged TG mice with advanced amyloid pathology and impaired microglial activity. Results showed a slight but significant improvement in the RI of microglia in Avasimibe-treated TG mice compared to WT (Fig. 7, Supplementary Movie 3).

**Fig. 8:**
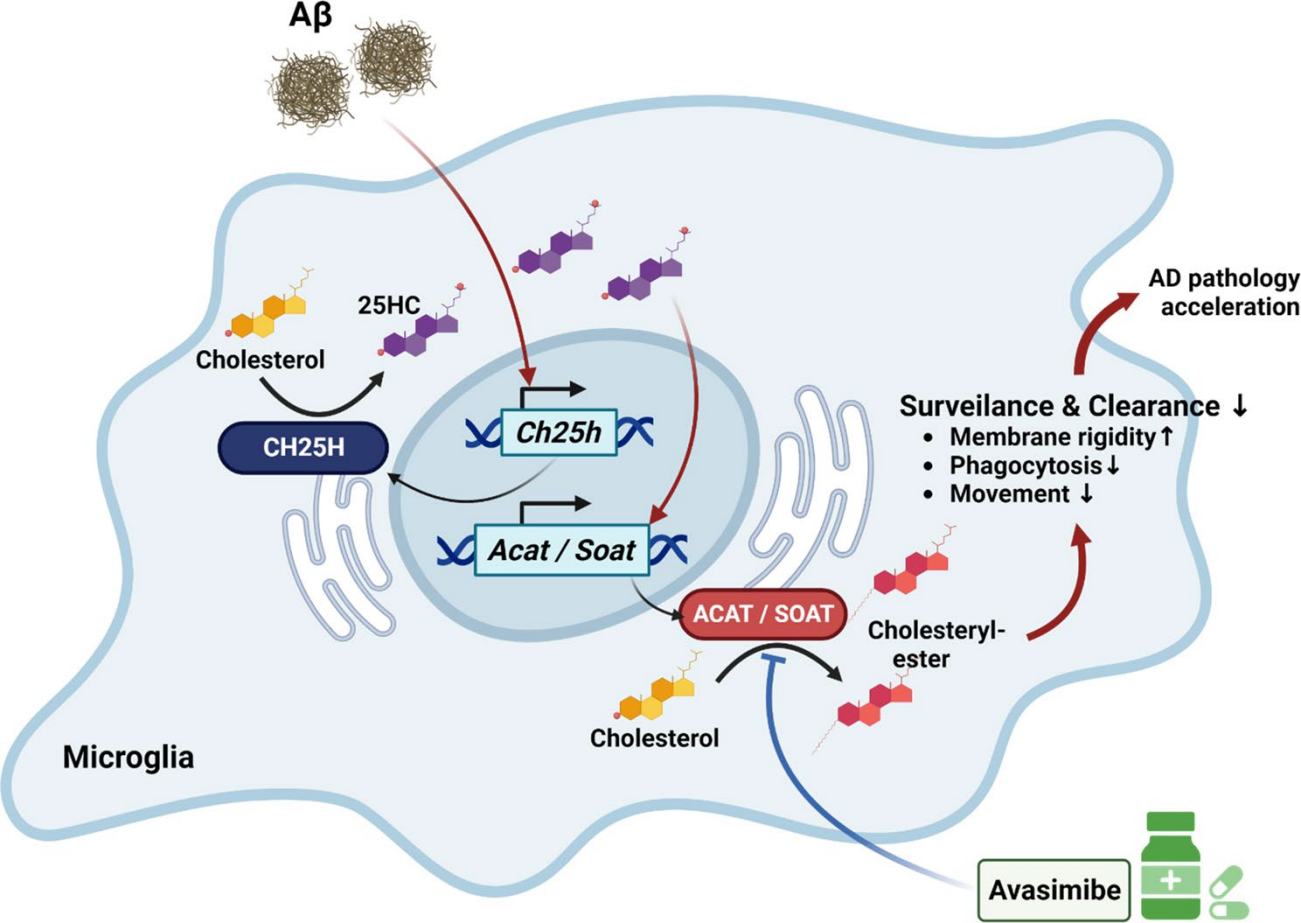
Graphical summary

These findings demonstrate that 25HC impairs microglial function, accelerates amyloid accumulation, and exacerbates AD-like cognitive and behavioral deficits. Importantly, Avasimibe treatment restores microglial function and ameliorates AD pathology (Fig. 8).

## Discussion

The findings of this study provide significant insights into the role of 25HC in modulating microglial function and its implications for AD pathology. Our data reveal that 25HC, a metabolite produced by the cholesterol hydroxylase encoded by the *Ch25h* gene, exerts profound effects on microglial surveillance, phagocytic activity, and cytokine production. Specifically, 25HC impairs the microglial response to brain injury, reduces their phagocytic capacity, and promotes a pro-inflammatory state factors that are all critical in the progression of AD. This study highlights that Aβ increases the expression of *Ch25h* in microglia, leading to elevated 25HC production, which in turn directly inhibits microglial movement and phagocytic capacity. One of the most striking observations is the significant reduction in microglial responsiveness to focal brain damage following 25HC administration, as demonstrated by *in vivo* two-photon microscopy. This diminished responsiveness correlates with a decrease in phagocytic capacity, observed both ex vivo and *in vitro*. These findings suggest that 25HC may contribute directly to microglial dysfunction in AD, where impaired phagocytosis of Aβ and other pathological debris is a hallmark. Moreover, the study highlights the role of Aβ in upregulating *Ch25h* expression and 25HC production in microglia, creating a feedback loop that could accelerate AD progression. Elevated CE levels and the resulting membrane rigidity likely impair microglial mobility and phagocytosis of Aβ, further contributing to the accumulation of amyloid plaques in the brain.

Mechanistically, our investigation reveals that 25HC stimulates cholesterol esterification, leading to disruptions in cell membrane dynamics. This finding aligns with previous reports that 25HC influences cholesterol metabolism in immune cells, particularly macrophages, by promoting cholesterol esterification and altering membrane composition [12, 13]. In 25HC-treated microglia, the observed increase in CE levels and the corresponding decrease in membrane fluidity provide a crucial mechanistic link between altered cholesterol metabolism and microglial dysfunction. The interplay between 25HC and the APOE protein isoforms also merits further investigation, as it has been reported that 25HC increases inflammatory cytokine production more in the presence of the APOE4 isoform than APOE3 [36]. When Aβ was treated in ApoE4 human-derived iMG, lipid droplet accumulation was increased, accompanied by elevated CH25H expression [37]. Furthermore, 25HC accumulation in the lysosome has been reported to induce metabolic reprogramming leading to enhanced immunosuppressive function in a study of immunosuppressive macrophages found in pan-tumours [10]. Given the correlation between the metabolic maladaptation of microglia observed in chronic AD brains [25, 38], it would be also meaningful to further validate that 25HC-induced tolerogenic microglia in AD pathogenesis.

Importantly, this study demonstrated that inhibiting cholesterol esterification with Avasimibe, a known CE inhibitor, can reverse the functional deficits in microglia induced by 25HC. Avasimibe treatment restored microglial membrane dynamics, phagocytic capacity, and responsiveness to injury, both *in vitro* and *in vivo*. This restoration was accompanied by a reduction in amyloid pathology and an improvement in cognitive function in 5XFAD mice, a well-established model of AD. Notably, Avasimibe is known to alleviate Aβ pathology by inhibiting the toxic Aβ production pathway, suggesting that its beneficial effects may be due to both the restoration of microglial function and the reduction in Aβ production [34, 39–41].

However, there are limitations associated with mouse models. The actual pathogenesis of AD is complex and involves not only Aβ but also various factors such as pathological tau and ApoE. Additionally, species-specific differences between mouse microglia and human microglia may limit the applicability of the findings to fully represent AD pathogenesis. Nonetheless, DAM-related gene profiles have been similarly observed in both 5XFAD mice and the brains of human AD patients [42]. Furthermore, APOE genotype-driven microglial gene expression profiles have shown comparable characteristics in APOE-FAD mice and iPSC-derived microglia [43] While the current study did not directly utilize human brain samples to address interspecies differences, we aimed to bridge this gap by confirming the role of 25HC in iPSC-derived microglia alongside our findings.

The potential interaction between microglia and surrounding cells, particularly astrocytes, in response to 25HC is also an important aspect of this study. 25HC released from microglia may accumulate lipid droplets in astrocytes, alter cholesterol reuptake, and affect ApoE release [44]. Given that lipid droplet-accumulating cells have an inflammatory phenotype and reduced capacity for normal lipid metabolism [45], this 25HC-mediated crosstalk between microglia and astrocytes likely has a synergistic adverse effect on AD pathology.

The broader implications of these findings suggest that 25HC could influence AD pathology through mechanisms beyond the brain. Since oxysterols, including 25HC, have good BBB permeability, increased systemic 25HC levels due to peripheral infections might also affect microglial function in the brain. Elevated *Ch25h* expression and 25HC production have been identified as defense mechanisms in macrophages during various viral infections [14–16], raising the possibility that systemic infections could exacerbate AD pathology through increased 25HC. Further research is warranted to explore how peripheral immune responses and 25HC interact with microglia and other brain cells, which could reveal new therapeutic targets for AD.

In summary, this study identifies 25HC as a critical modulator of microglial function in AD, linking cholesterol metabolism to microglial dysfunction and disease progression. The efficacy of Avasimibe in reversing these effects underscores the potential of targeting cholesterol metabolism as a therapeutic strategy. Future studies should further investigate the direct regulation of 25HC through KO experiments, the role of other oxysterols, the interaction between 25HC and peripheral immune cells, and the potential for combination therapies that address multiple aspects of microglial dysfunction in AD.

## Electronic supplementary material

Below is the link to the electronic supplementary material.

Supplementary Material 1

Supplementary Material 2

Supplementary Material 3

Supplementary Material 4

Supplementary Material 5

## Data Availability

No datasets were generated or analysed during the current study.
