# Supplementary material for: 25-Hydroxycholesterol modulates microglial function and exacerbates Alzheimer’s disease pathology: mechanistic insights and therapeutic potential of cholesterol esterification inhibition
Source: J Neuroinflammation. 2025 Feb 25;22:50. doi: 10.1186/s12974-025-03357-y (PMC11863767; doi:10.1186/s12974-025-03357-y)

Figure 2D

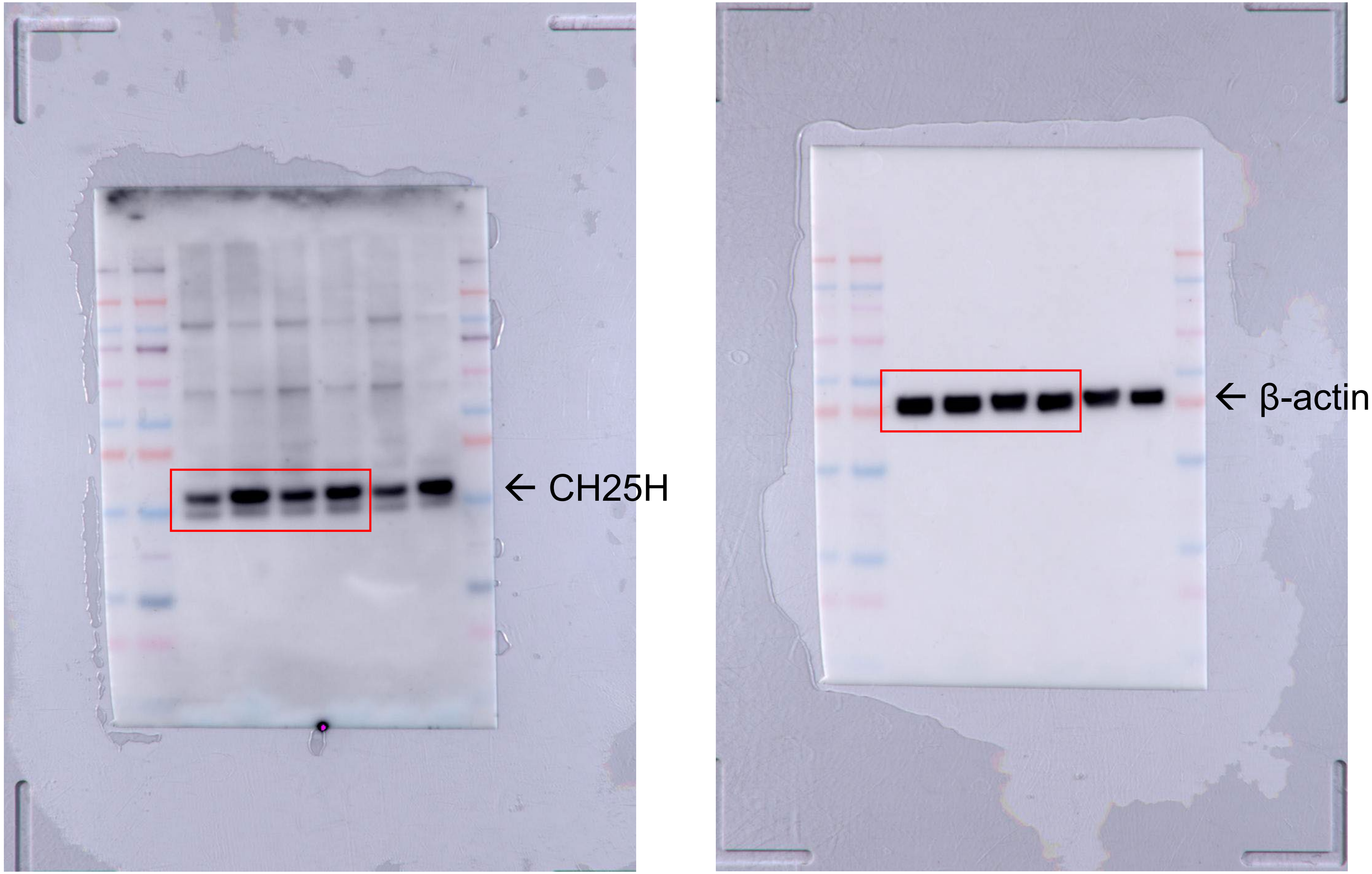

Extended Data Figure 2C

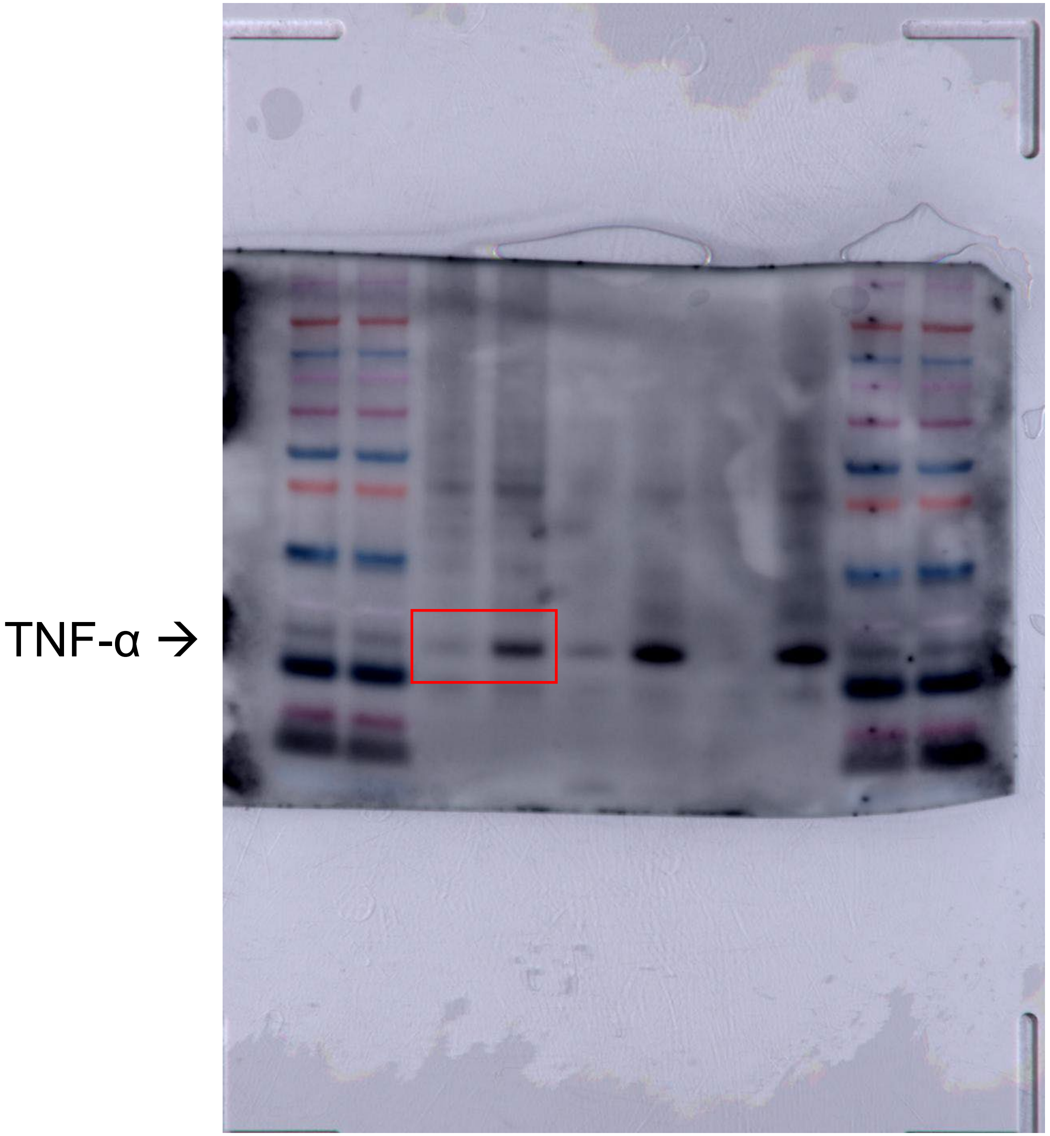

Extended Data Figure 2F

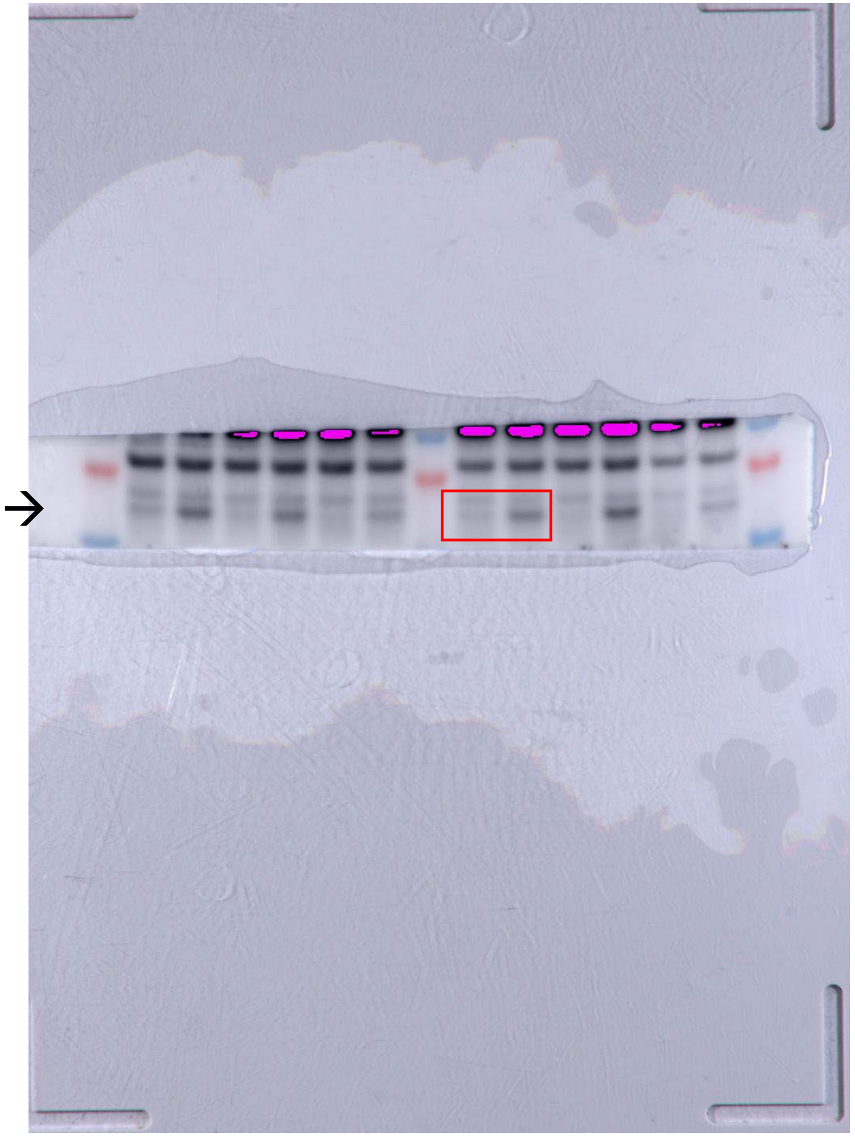

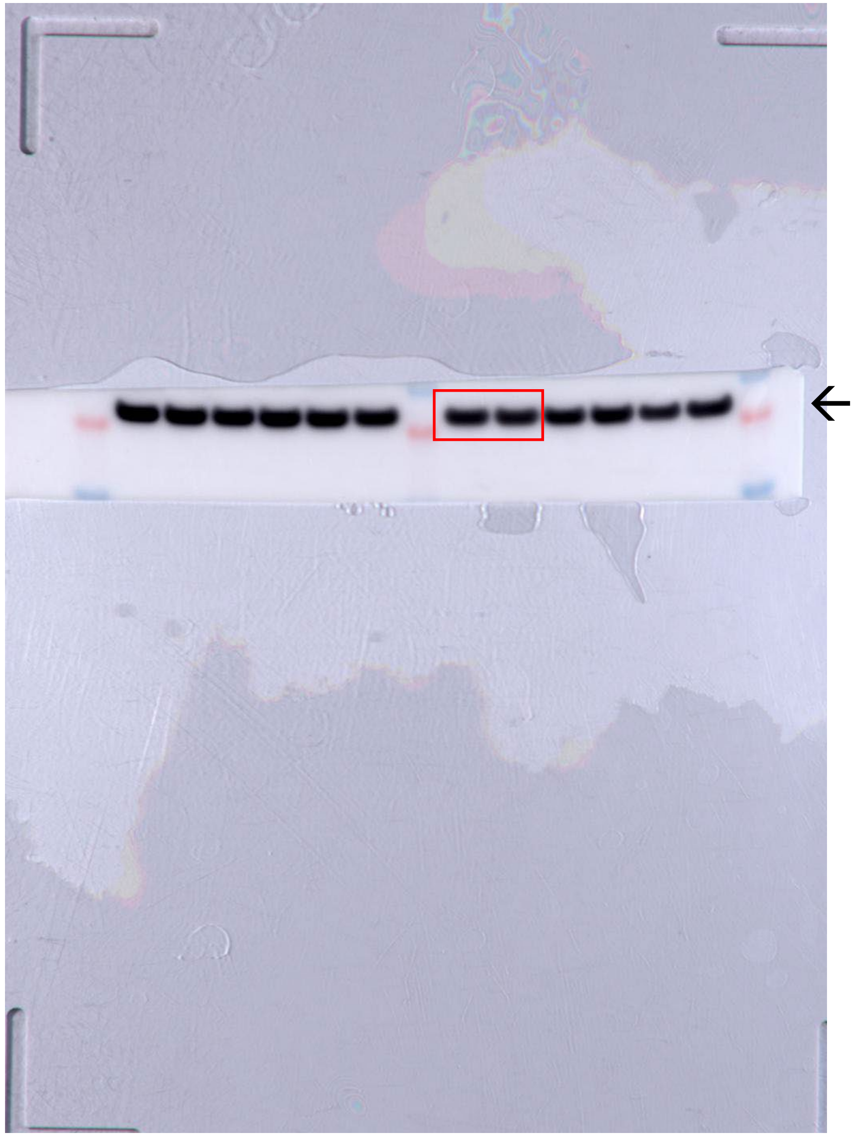

Extended Data Figure 7A

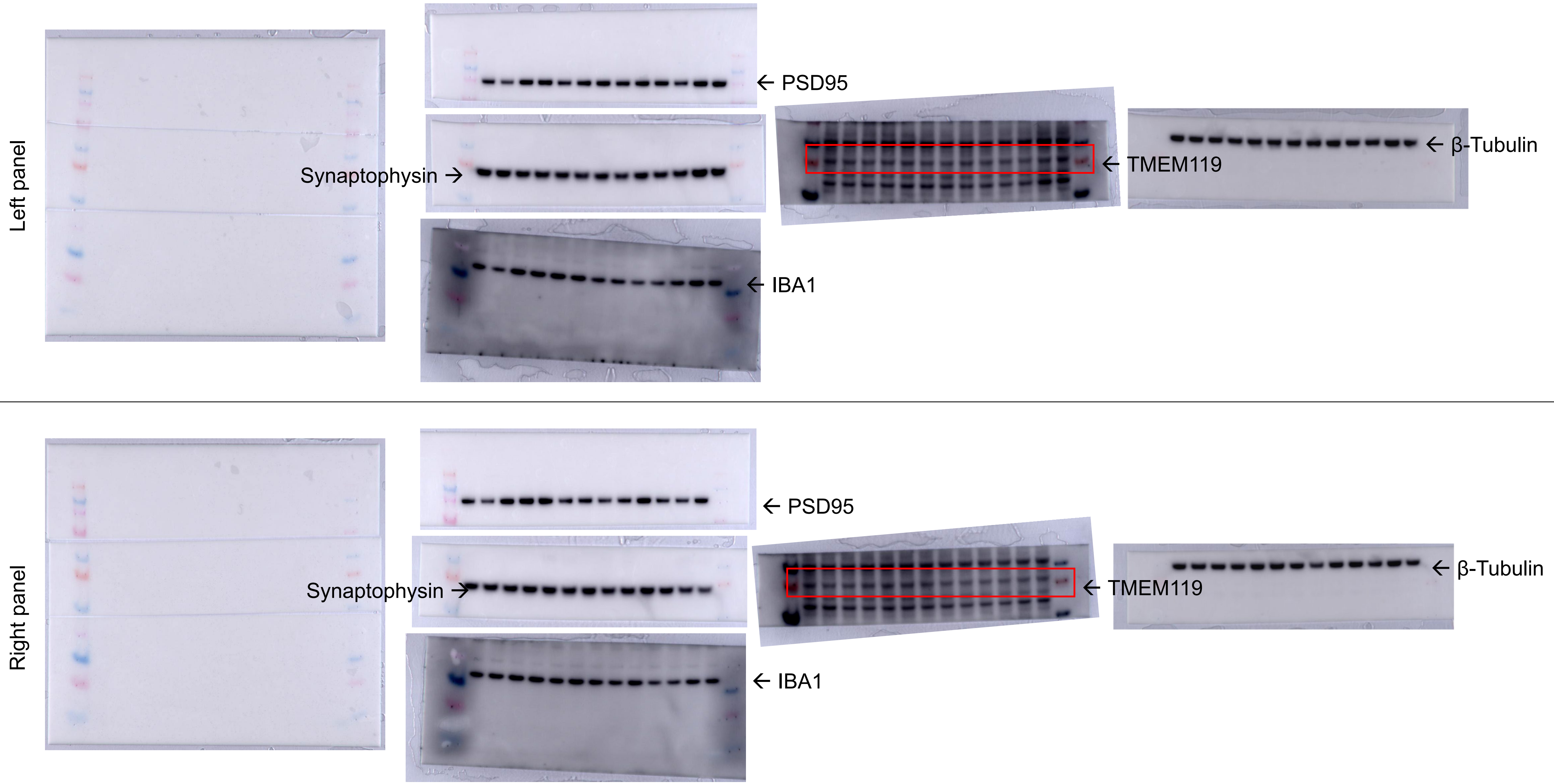

Supplement: Supplementary file 4 — Supplementary Material 4 [file 12974_2025_3357_MOESM4_ESM.pdf]
