# Supplementary material for: 25-Hydroxycholesterol modulates microglial function and exacerbates Alzheimer’s disease pathology: mechanistic insights and therapeutic potential of cholesterol esterification inhibition
Source: J Neuroinflammation. 2025 Feb 25;22:50. doi: 10.1186/s12974-025-03357-y (PMC11863767; doi:10.1186/s12974-025-03357-y)
Supplement: Supplementary file 5 — Supplementary Material 5 [file 12974_2025_3357_MOESM5_ESM.docx]

**25-Hydroxycholesterol Modulates Microglial Function and Exacerbates Alzheimer's Disease Pathology: Mechanistic Insights and Therapeutic Potential of Cholesterol Esterification Inhibition**

Hayoung Choi^1,2,^ **^†^**, Haeng Jun Kim^2,^ **^†^**, Sang-Eun Lee^3^, Hyun Ho Song^4^, Jieun Kim^1^, Jihui Han^1^, June-Hyun Jeong^1^, Do Yup Lee^4^, Sunghoe Chang^3^, Inhee Mook-Jung^1,2,^*

**^†^These authors contributed equally to this work.**

**Author affiliations:**

1 Department of Biochemistry and Biomedical Sciences, College of Medicine, Seoul National University, Seoul 03080, Republic of Korea

2 Convergence Dementia Research Center, Medical Research Center, Seoul National University, Seoul 03080, Republic of Korea

3 Department of Physiology and Biomedical Sciences, College of Medicine, Seoul National University, Seoul 03080, Republic of Korea

4 Department of Agricultural Biotechnology, Center for Food and Bioconvergence, Research Institute for Agricultural and Life Sciences, Seoul National University, Seoul 08826, Republic of Korea

Correspondence to: Inhee Mook-Jung

Full address: Department of Biochemistry and Biomedical Sciences, College of Medicine, Seoul National University, Seoul 03080, Republic of Korea

E-mail: inhee@snu.ac.kr

**Supplementary Information for**

**25-Hydroxycholesterol Modulates Microglial Function and Exacerbates Alzheimer's Disease Pathology: Mechanistic Insights and Therapeutic Potential of Cholesterol Esterification Inhibition**

**Contents**

Materials

Extended figures and figure legends

**Materials**

**The primer information corresponding to each target mRNA used in the study**

mouse Rps18 F: 5’-GGATGTGAAGGATGGGAAGT-3’

mouse Rps18 R: 5’-CCCTCTATGGGCTCGAATTT-3’

mouse Ch25h F: 5’-TGCTACAACGGTTCGGAGC-3’

mouse Ch25h R: 5’-AGAAGCCCACGTAAGTGATGAT-3’

mouse Acat1 F: 5’-CAGGAAGTAAGATGCCTGGAAC-3’

mouse Acat1 R: 5’-TTCACCCCCTTGGATGACATT-3’

mouse Acat2 F: 5’-AGACTTGGTGCAATGGACTCGAC-3’

mouse Acat2 R: 5’-CATAGGGCCCGATCCAACAG-3’

mouse Soat F: 5’-GAAACCGGCTGTCAAAATCTGG-3’

mouse Soat R: 5’-TGTGACCATTTCTGTATGTGTCC-3’

**The primary antibody information used in the study**

| Antibody | Company | #Catalog | Application | Titer |
| --- | --- | --- | --- | --- |
| Mouse monoclonal anti-β-Amyloid,  biotin-labeled, clone: 4G8 | BioLegend | 800704 | IHC | 1:1000 |
| Anti Iba1 | FUJIFILM Wako Pure Chemical Corporation | 019-19741 | IHC | 1:500 |
|  |  |  | RNA-ISH | 1:20 |
|  |  |  | WB | 1:2000 |
| IBA1 antibody/ Guinea pig monoclonal recombinant IgG | SYSY | #234 408 | IHC | 1:500 |
| GFAP monoclonal antibody2.2B10 | Invitrogen | 13-0300 | IHC | 1:500 |
|  |  |  | RNA-ISH | 1:50 |
| NeuN | Cell signaling technology | 24307 | IHC | 1:500 |
| CH25H Polyclonal Antibody | Invitrogen | PA5-72349 | WB | 1:2000 |
| Mouse monoclonal anti-β-actin | Sigma-Aldrich | Ab1978 | WB | 1:2000 |
| Goat polyclonal anti-mouse Il-1β | R&D systems | AF-401-NA | WB | 1:2000 |
| Human/Mouse TNF-α antibody | R&D systems | AF-410-NA | WB | 1:2000 |
| Anti-PSD95 antibody | Abcam | Ab18258 | WB | 1:2000 |
| Anti-Synaptophysin antibody | MilliporeSigma | MAB368 | WB | 1:2000 |
| Anti-TMEM119 antibody [28-3] | Abcam | Ab209064 | WB | 1:2000 |
| Monoclonal Anti-β-Tubulin I+II antibody produced in mouse | Sigma-Aldrich | T8535 | WB | 1:2000 |
| Rat monoclonal anti-CD11b, APC | eBioscience | 17-0112-81 | FACS | 1:1000 |
| PE/Cy7 anti-mouse CD45.2 Antibody | Biolegend | 109829 | FACS | 1:500 |

**Extended figures and figure legends**

**Extended Data Fig. 1: Aβ significantly increases Ch25h only in microglia among cells in the brain**

**(A, B)** Representative image of 5XFAD_TG brain tissue labeled with *Ch25h* by RNA-ISH technique co-stained with antibodies against IBA1 or GFAP (Magnified image scale bar = 25 μm; Scale bar = 20 μm).

**(C-E)** Ch25h mRNA expression level after treating mouse PMG, primary astrocytes, and primary neuron with vehicle or Aβ (4 μM) or LPS (10 ng/ml) for 24 hours (7-month-old; *N=4-5*; Mean ± SEM; One-way ANOVA, Tukey's multiple comparisons; ***p<0.01, ****p<0.001, ns, not significant*).

**Extended Data Fig. 2: Bead uptake capacity of isolated 5XFAD adult microglia and the gating strategy for bead uptake analysis**

**(A)** Ex vivo isolated microglia bead uptake FACS analysis (*N=3*; Mean ± SEM; One-way ANOVA, Tukey's multiple comparisons; **p<0.05*).

**(B, C)** Gating strategy for analyzing bead uptake microglia

**Extended Data Fig. 3: 25HC increases microglial proinflammatory cytokines expression and secretion**

**(A, D)** Tnf-α and Il-1β mRNA expression level after treating PMG with vehicle or 25HC (5 μg/ml) for 24 hours (*N=5-7*).

**(B, C)** TCA precipitated TNF-α protein level in the culture medium of PMG treated with vehicle or 25HC (5 μg/ml) for 24 hours quantification by WB (*N=4*).

**(E, F)** The IL-1β protein expression level was measured by WB after treatment of PMG with vehicle or 25HC (5 μg/ml) for 24 hours (*N=5*).

(Mean ± SEM; unpaired t-test; **p<0.05, **p<0.01*).

**Extended Data Fig. 4: 25HC and Aβ stimulate cholesterol esterification, disrupting membrane dynamics in PMG, and Avasimibe restores them**

**(A, B)** Acat1 and Soat1 mRNA expression level after treating PMG with Vehicle or Aβ (4 μM) or 25HC (5 μg/ml) for 24 hours (*N=6-7*; Tukey's multiple comparisons)

**(C-E)** Cholesterol and cholesteryl ester quantification graph of all groups related to Fig. 3-4 (*N=4*; Tukey's multiple comparison tests).

**(F-H)** Quantification of mobile fraction and τ of FRAP analysis of all groups related to Fig. 3-4 (*N=4*; Holm-Sidak multiple comparison test).

 (Mean ± SEM; *N=6-7*; One-way ANOVA, Tukey's multiple comparisons; **p<0.05, **p<0.01, ****p<0.001*)

**Extended Data Fig. 5: Locomotor behavior analysis of 5XFAD mice after 25HC and Avasimibe treatment**

**(A)** The total entry number of the mouse entered at the arm to evaluate the basic movement level during the Y-MAZE (*N=19-22*).

**(B, C)** Quantitative graph of the mouse total distance moved by open field test and representative visualized track images (*N=13-18*).

**(D)** Changes in mouse body weight before and after drug administration

**(E)** Representative visualized heatmap images of NOR test

Extended Data Fig. 6: Aβ pathology analysis of 5XFAD_TG mice after 25HC and Avasimibe treatment

**(A-D)** Representative images and quantitative results of 5XFAD mouse hippocampus observed with 4G8, CongoRed, and IBA1(*N=8-10*, Tukey's multiple comparisons).

**(E, F)** Quantitative results of RIPA soluble and insoluble Aβ_1-42_ present in mouse hippocampus by ELISA (*N=9-10*; Hols-Sidak's multiple comparison test).

**(G-I)** Representative images and quantitative results of 5XFAD mouse brain hemisphere (tile images) observed with 4G8, CongoRed (*N=8-11*, Tukey's multiple comparisons).

 (*N=8-10*; Mean ± SEM; unpaired *t* test; *#p<0.05*; One-way ANOVA; **p<0.05, ns, non-significant*)

**Extended Data Fig. 7: Glial and neuronal changes in 5XFAD_TG mice after treatment with 25HC and Avasimibe**

**(A-E)** The representative blot images and quantification of protein level of IBA1, TMEM119, Synaptophysin, and PSD95 in mouse hippocampus measured by WB (*N=11-15*, One-way ANOVA)

**(F-K)** Representative images and quantitative data of 5XFAD mouse cerebral cortex were observed with GFAP and NeuN(A-C) Representative images and quantitative data of 5XFAD mouse hippocampus observed with GFAP and NeuN

(Mean ± SEM; *N=8-10*; One-way ANOVA, Tukey's multiple comparison tests; ***p<0.01*).

**S. video 1. In vivo microglial response after focal laser ablation of CX3CR1^GFP/+^_WT mice after 25HC treatment**

**S. video 2. In vivo microglial response after focal laser ablation of CX3CR1^GFP/+^_ WT mice after 25HC and Avsimibe treatment**

**S. video 3. In vivo microglial response after focal laser ablation of CX3CR1^GFP/+^_5XFAD mice after 25HC and Avasimibe treatment**


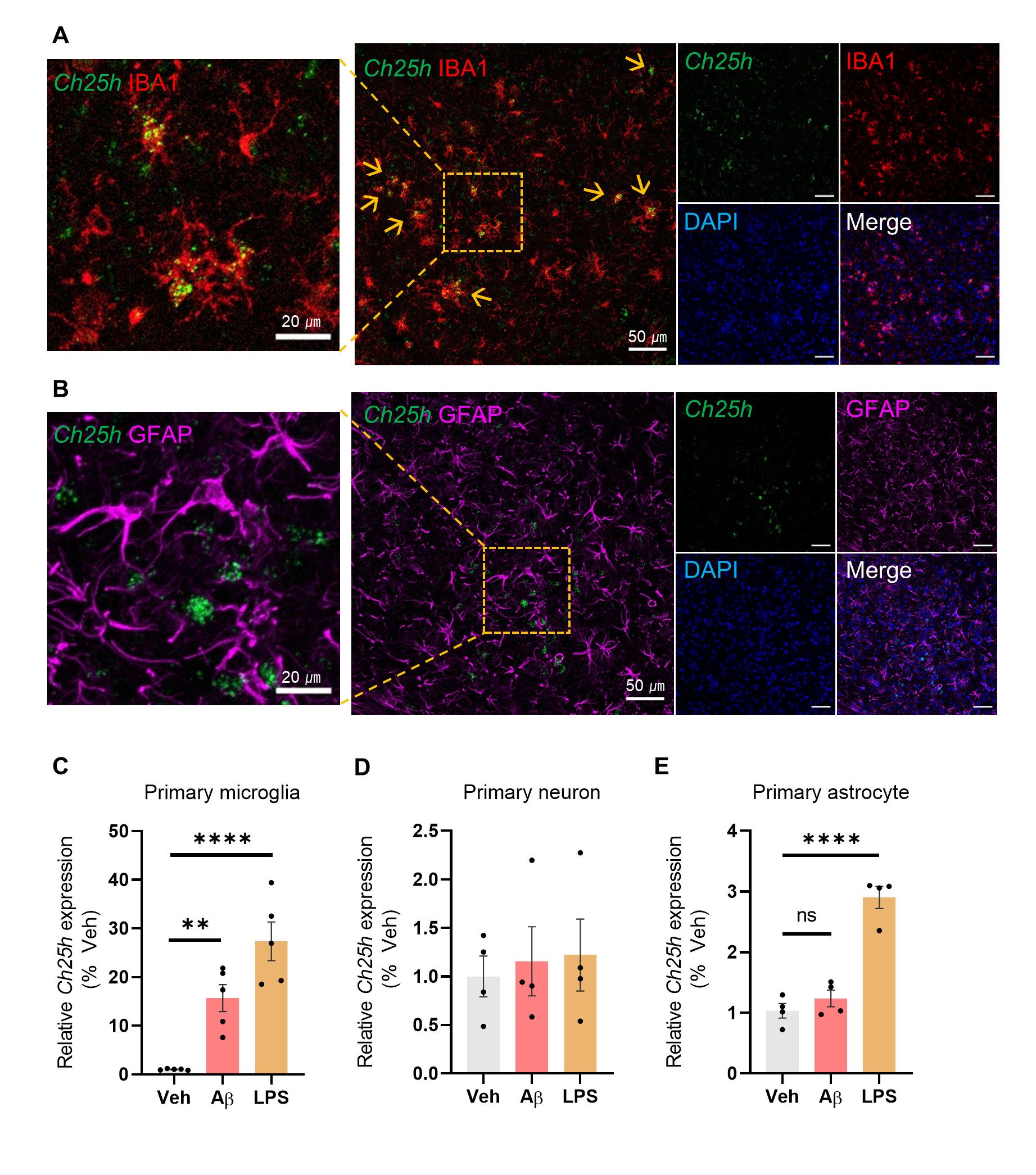


Extended Data Fig. 1: Aβ significantly increases *Ch25h* only in microglia among cells in the brain


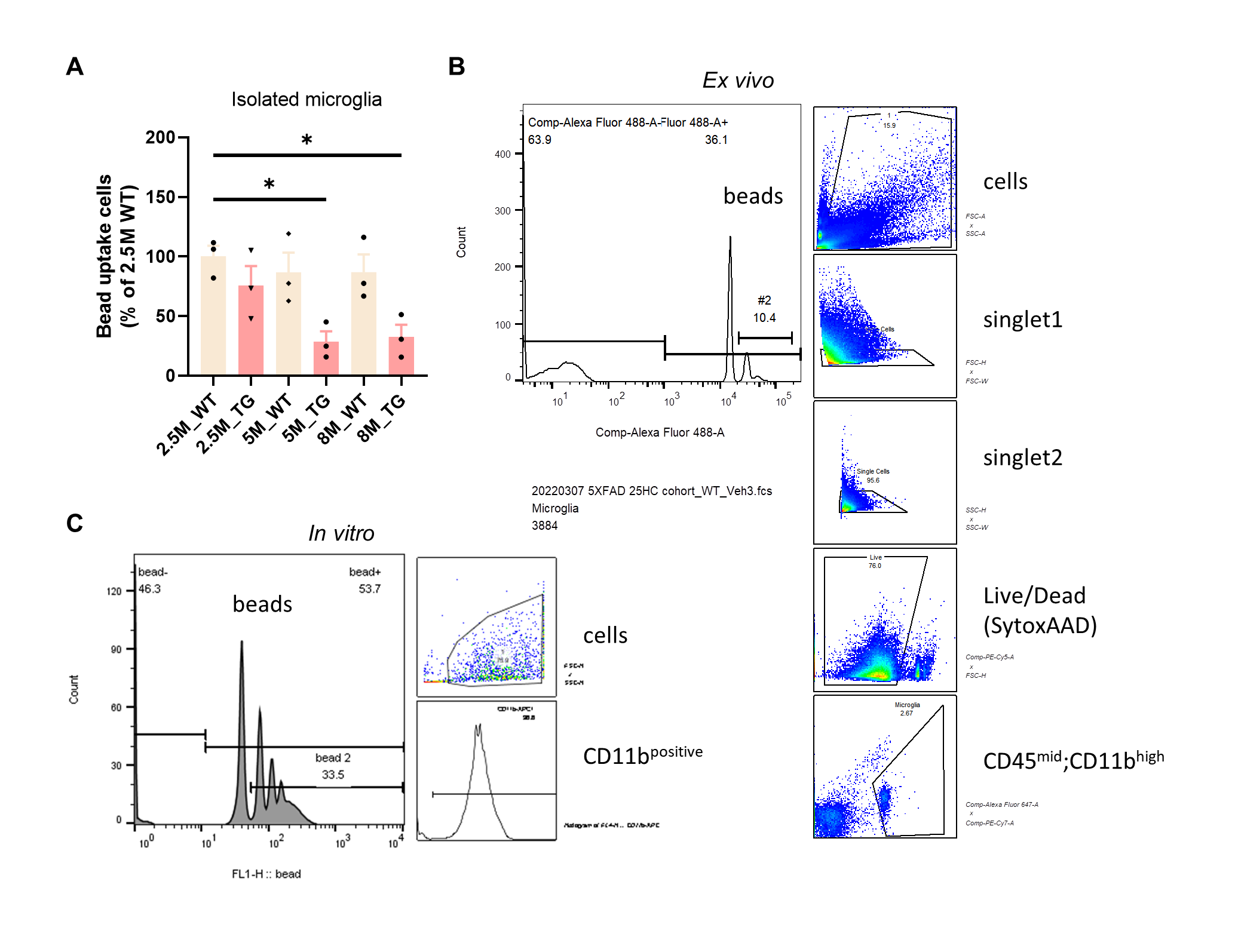


Extended Data Fig. 2: Bead uptake capacity of isolated 5XFAD adult microglia and the gating strategy for bead uptake analysis


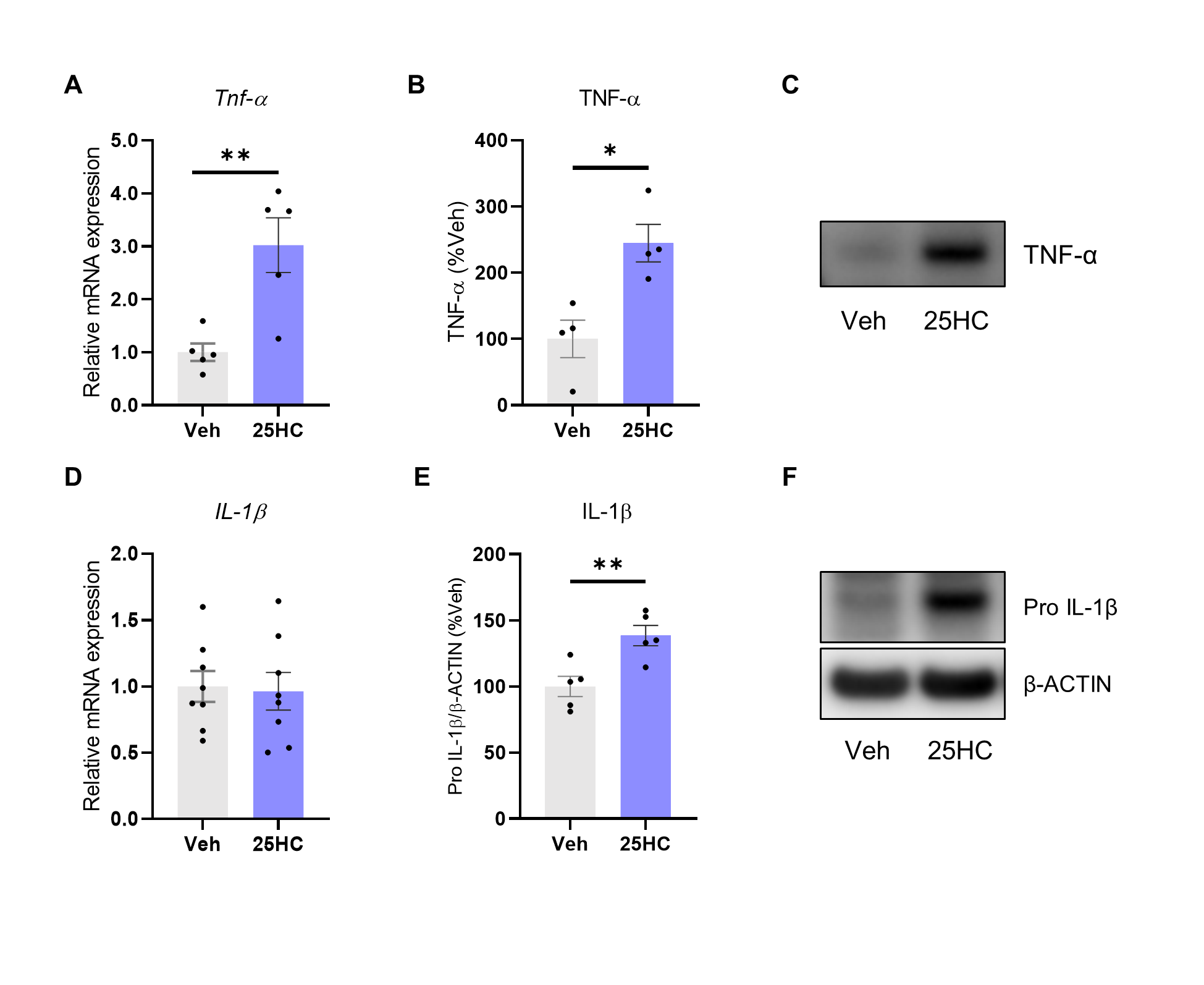


Extended Data Fig. 3: 25HC increases microglial proinflammatory cytokines expression and secretion


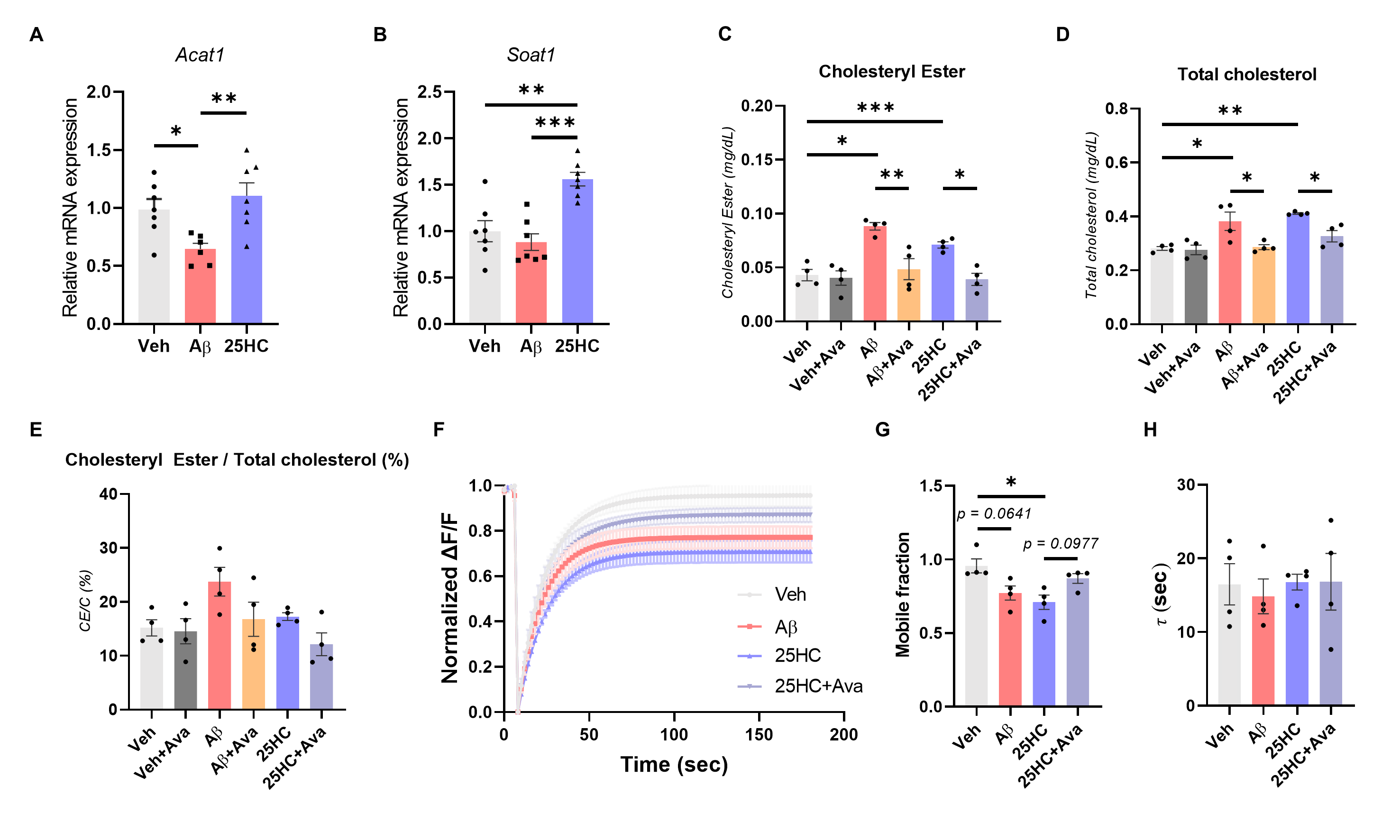


Extended Data Fig. 4: 25HC and Aβ stimulate cholesterol esterification, disrupting membrane dynamics in PMG, and Avasimibe restores them

**Extended Data Fig. 5: Locomotor behavior analysis of 5XFAD_TG mice after 25HC and Avasimibe treatment**


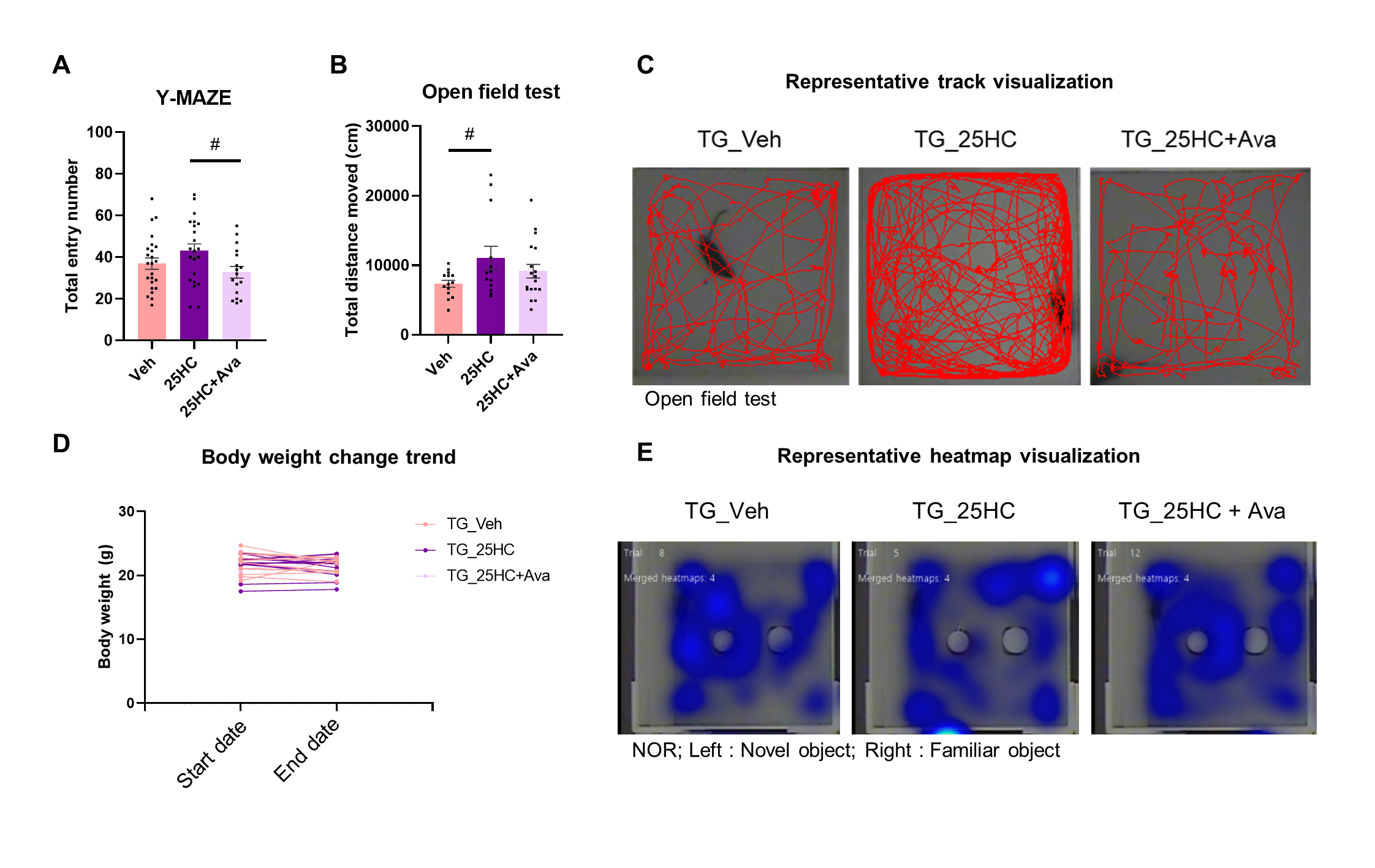


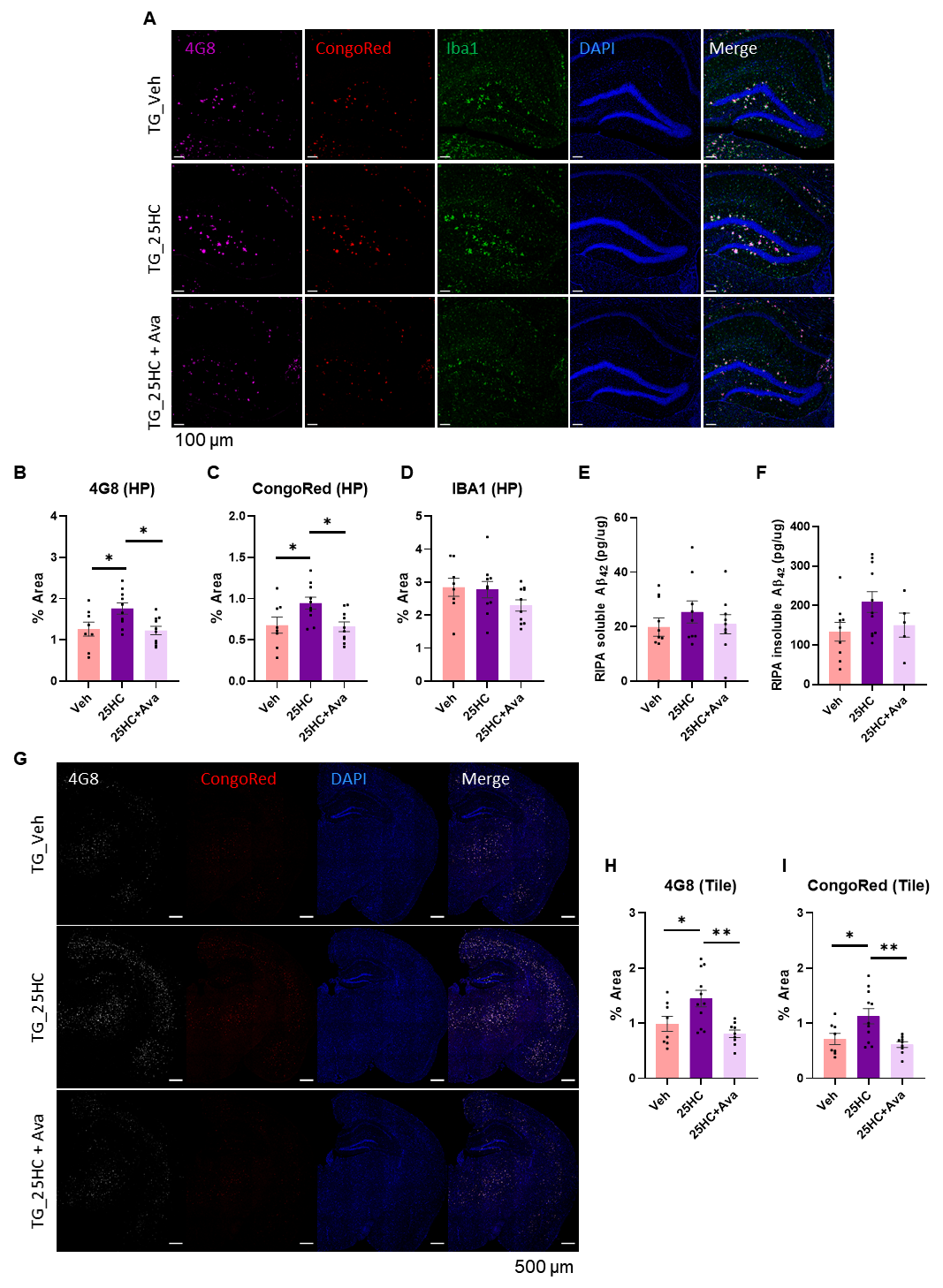


Extended Data Fig. 6: Aβ pathology analysis of 5XFAD_TG mice after 25HC and Avasimibe treatment


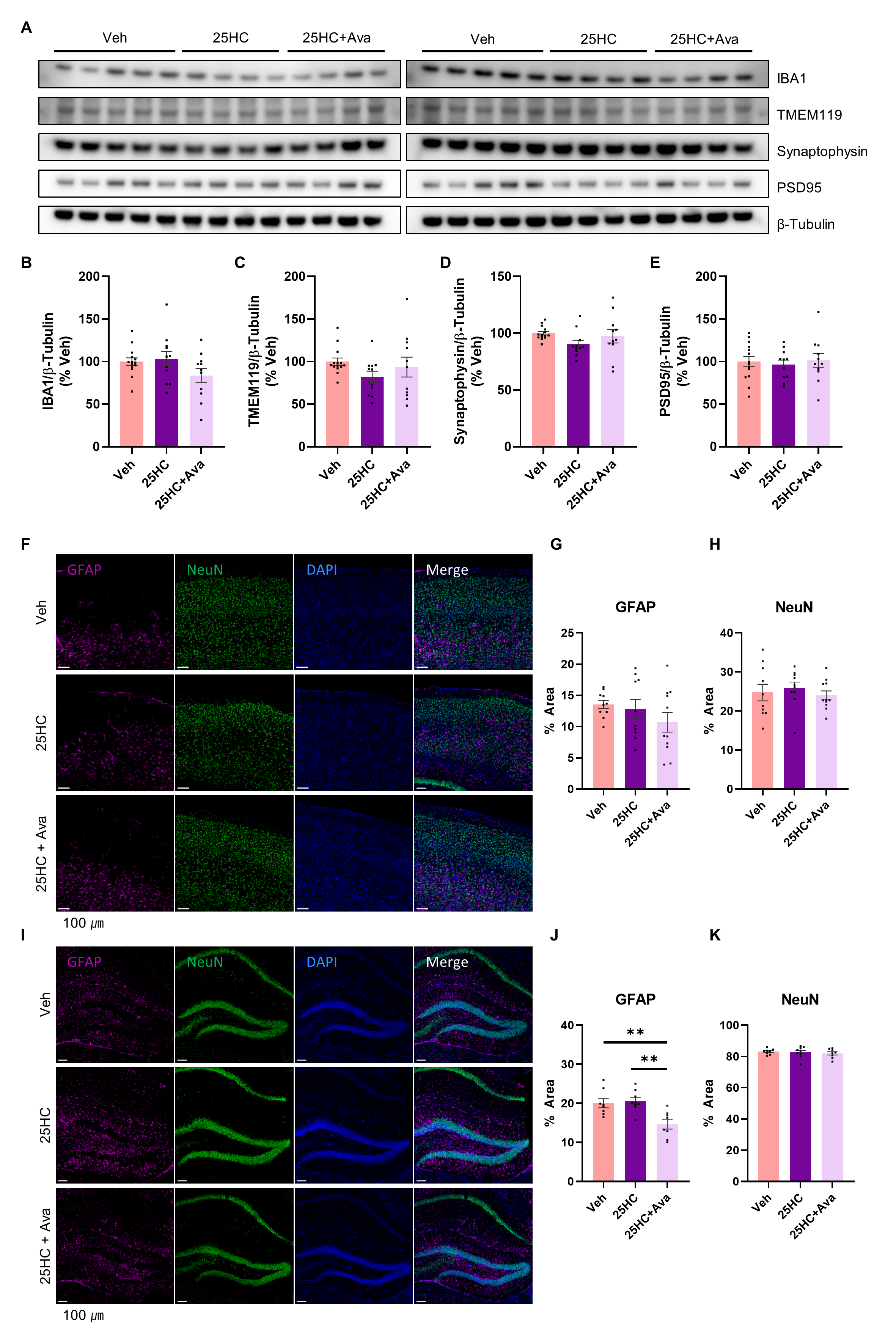


Extended Data Fig. 7: Glial and neuronal changes in 5XFAD_TG mice after treatment with 25HC and Avasimibe
